# PAND: A Distribution to Identify Functional Linkage from Networks with Preferential Attachment Property

**DOI:** 10.1371/journal.pone.0127968

**Published:** 2015-07-09

**Authors:** Hua Li, Pan Tong, Juan Gallegos, Emily Dimmer, Guoshuai Cai, Jeffrey J. Molldrem, Shoudan Liang

**Affiliations:** 1 Bio-ID Center, School of Biomedical Engineering, Shanghai Jiao Tong University, Shanghai, 200240, China; 2 Department of Stem Cell Transplantation and Cellular Therapy, The University of Texas MD Anderson Cancer Center, Houston, Texas, 77030, United States of America; 3 Department of Bioinformatics and Computational Biology, The University of Texas MD Anderson Cancer Center, Houston, Texas, 77030, United States of America; 4 Department of Molecular and Human Genetics, Baylor College of Medicine, Houston, Texas, 77030, United States of America; 5 The EMBL Outstation-European Bioinformatics Institute, Wellcome Trust Genome Campus, Hinxton, Cambridge, CB10 1SD, United Kingdom; Universitat Pompeu Fabra, Barcelona Research Park of Biomedicine (PRBB), SPAIN

## Abstract

Technology advances have immensely accelerated large-scale mapping of biological networks, which necessitates the development of accurate and powerful network-based algorithms to make functional inferences. A prevailing approach is to leverage functions of neighboring nodes to predict unknown molecular function. However, existing neighbor-based algorithms have ignored the scale-free property hidden in many biological networks. By assuming that neighbor sharing is constrained by the preferential attachment property, we developed a Preferential Attachment based common Neighbor Distribution (PAND) to calculate the probability of the neighbor-sharing event between any two nodes in scale-free networks, which nearly perfectly matched the observed probability in simulations. By applying PAND to a human protein-protein interaction (PPI) network, we showed that smaller probabilities represented closer functional linkages between proteins. With the PAND-derive linkages, we were able to build new networks where the links are more functionally reliable than those of the human PPI network. We then applied simple annotation schemes to a PAND-derived network to make reliable functional predictions for proteins. We also developed an R package called *PANDA* (PAND-derived functional Associations) to implement the methods proposed in this study. In conclusion, PAND is a useful distribution to calculate the probability of the neighbor-sharing events in scale-free networks. With PAND, we are able to extract reliable functional linkages from real biological networks and builds new networks that are better bases for further functional inference.

## Introduction

High-throughput screenings have been generating massive amount of biological data at an unprecedented speed. From genomic sequence to epigenetic modification, from gene expression to protein-protein interaction (PPI), the accumulation of various types of data leads to the rapid discovery of new cellular components, such as new proteins and non-coding RNAs (ncRNAs). However, a considerable portion of these components has yet to be functionally characterized. For example, even for the well-studied model organism *Schizosaccharomyces pombe*, the functions of over 900 genes remain unknown [1]. The situation is more severe in mammals because they have more genes and many genes have multiple functions. Fortunately, recent development of computational methods based on the characteristics of large biological networks has made it possible to infer the biological functions of network components on a global scale [2–8]. For example, the neighbor-based methods infer a protein’s function based on its immediate neighborhood [9–15], while the graph theoretic methods use the global topology of a network to make functional inference [2,4,16].

Biological networks can be abstracted using simplified graphs with nodes representing cellular components and links representing interactions between them. Based on the assumption that neighboring nodes in networks tend to share similar biological functions, previous works have developed various statistical techniques to make functional predictions for cellular components [7]. In PPI networks, for example, Schwikowski *et al* (2000) annotated a protein according to the most prevalent function(s) among its direct neighbors in the network; Hishigaki *et al* (2001) proposed a *χ* ^2^ statistic to predict protein functions based on that of neighbors lying within a certain radius; and Li and Liang (2009) used information on common neighbors to perform functional annotation and clustering. Although these neighbor-based studies have shown excellent performance and yielded a handful of predictions, none of them has incorporated the topological property of scale-free network that has been well established for many biological networks, social networks, the Internet, etc. [17]. Inspired by the Barabasi-Albert model [18–19], we assume that a scale-free network has the following preferential attachment (PA) property: a node with a larger degree (degree is the number of links attached to any node in a network) is more likely to be connected by other nodes in the network. This assumed PA property reflects the difference between nodes in scale-free networks [3,17], and necessitates treating nodes unequally when developing neighbor-based statistical models. For example, in Samanta and Liang (2003), the probability of the neighbor-sharing events needs to be re-estimated since the basic assumption (i.e., each node has the same probability to be picked by a given node as its neighbor) is not appropriate in a network with the PA property.

In this study, we developed a Preferential Attachment based common Neighbor Distribution (PAND) to calculate the probability of two nodes sharing a certain number of common neighbors in scale-free networks. When deriving PAND, we weighted each node based on the assumption that the probability of connecting an existing node is linearly proportional to its degree. Compared with a previous work without PA assumption [11], PAND immensely improved the probability estimation of the neighbor-sharing events in randomized scale-free networks. As each link in a biological network (defined as a direct link) is also informative on the functional association between two nodes, we further incorporated this information into PAND by converting a direct link into λ common neighbors (*λ* ≥ 0). Based on a real human PPI network, we showed that PAND revealed higher-quality functional links between proteins than the previous work [11] (We used the Gene Ontology (GO) and Kyoto Encyclopedia of Genes and Genomes (KEGG) databases to assess the quality of the derived links [20–21]). Based on these links, we were able to build a new network and employ existing direct and module-assisted annotation schemes to make reliable functional predictions [7]. In addition, we developed an R package called *PANDA* (PAND-derived functional Associations) to easily apply the PAND distribution for functional inference.

## Results

### Preferential Attachment based common Neighbor Distribution (PAND)

Samanta and Liang (2003) developed a statistical model to calculate the probability of the neighbor-sharing events and showed that a very small probability indicates a close functional relationship between two nodes [11]. Here we develop a new model as follows to calculate the probability of the same events in scale-free networks. In a network with a total of *n* nodes, suppose we add two new nodes: *A* and *B*, with *k*_*A*_ as the degree of node *A* and *k*_*B*_ as the degree of node *B*. Assuming that the preferential attachment (PA) probability of connecting an existing node is linearly proportional to its degree, we derived the following formula for calculating the probability that two nodes (*A* and *B*) share *m* common neighbors in scale-free networks (see Materials and Methods for details):

$$P_{S}\left(m|k_{A},k_{B},n\right)=\frac{\phi \left(\begin{array}{c} n\\ m \end{array}\right)\left(\begin{array}{c} n-m\\ k_{A}-m \end{array}\right)\left(\begin{array}{c} n-k_{A}\\ k_{B}-m \end{array}\right){\left[E\left(K^{2}\right)\right]}^{m}}{\left(\begin{array}{c} n\\ k_{A} \end{array}\right)\left(\begin{array}{c} n\\ k_{B} \end{array}\right){\left[E\left(K\right)\right]}^{2m}}$$ (1)

In formula (1), subscript *“S”* denotes preferential attachment, *K* denotes the degree, *E*(*K*) is the average degree of the network [it is considered as a constant in formula (1)] and *ϕ* is the normalizing constant. Thus, *E*(*K* ^2^) − [*E*(*K*)]^2^ = *Var*(*K*). In a scale-free network, because of the relative commonness of high-degree (i.e., hubs) and low-degree nodes, *Var*(*K*) is large enough to make a difference between *E*(*K* ^2^) and [*E*(*K*)]^2^. Therefore, as *m* increases, [*E*(*K* ^2^)]^*m*^ becomes much larger than [*E*(*K*)]^2*m*^. However, in the simple random network proposed by Erdos and Renyi [22], it is rare to observe nodes with degrees that are much larger or smaller than the average degree of the network. As a result, *Var*(*K*) ≪ *E*(*K* ^2^), *E*(*K* ^2^) ≈ [*E*(*K*)]^2^, and [*E*(*K* ^2^)]^*m*^ ≈ [*E*(*K*)]^2*m*^. Moreover, if *k*_A_*k_*B*_ ≪ *n*, *ϕ* will be close to 1. Therefore, in a simple network with [*E*(*K*)]^2^ ≪ *n*, formula (1) approximates the one proposed by Samanta and Liang (2003) [11]:

$$P\left(m|k_{A},k_{B},n\right)=\frac{\left(\begin{array}{c} n\\ m \end{array}\right)\left(\begin{array}{c} n-m\\ k_{A}-m \end{array}\right)\left(\begin{array}{c} n-k_{A}\\ k_{B}-m \end{array}\right)}{\left(\begin{array}{c} n\\ k_{A} \end{array}\right)\left(\begin{array}{c} n\\ k_{B} \end{array}\right)}$$ (2)

Compared with formula (2), formula (1) integrates the information of the degree variance in the network. The additional terms of formula (1) indicate that the events of sharing a large number of common neighbors are more readily observed in scale-free networks than in simple random networks, which is in accordance with our simulation results in the following paragraph. A flowchart of our work is shown in S1 Fig to describe the important steps in this study and the logical relationship between them.

### Simulation-based analysis of PAND

**Fig 1:**
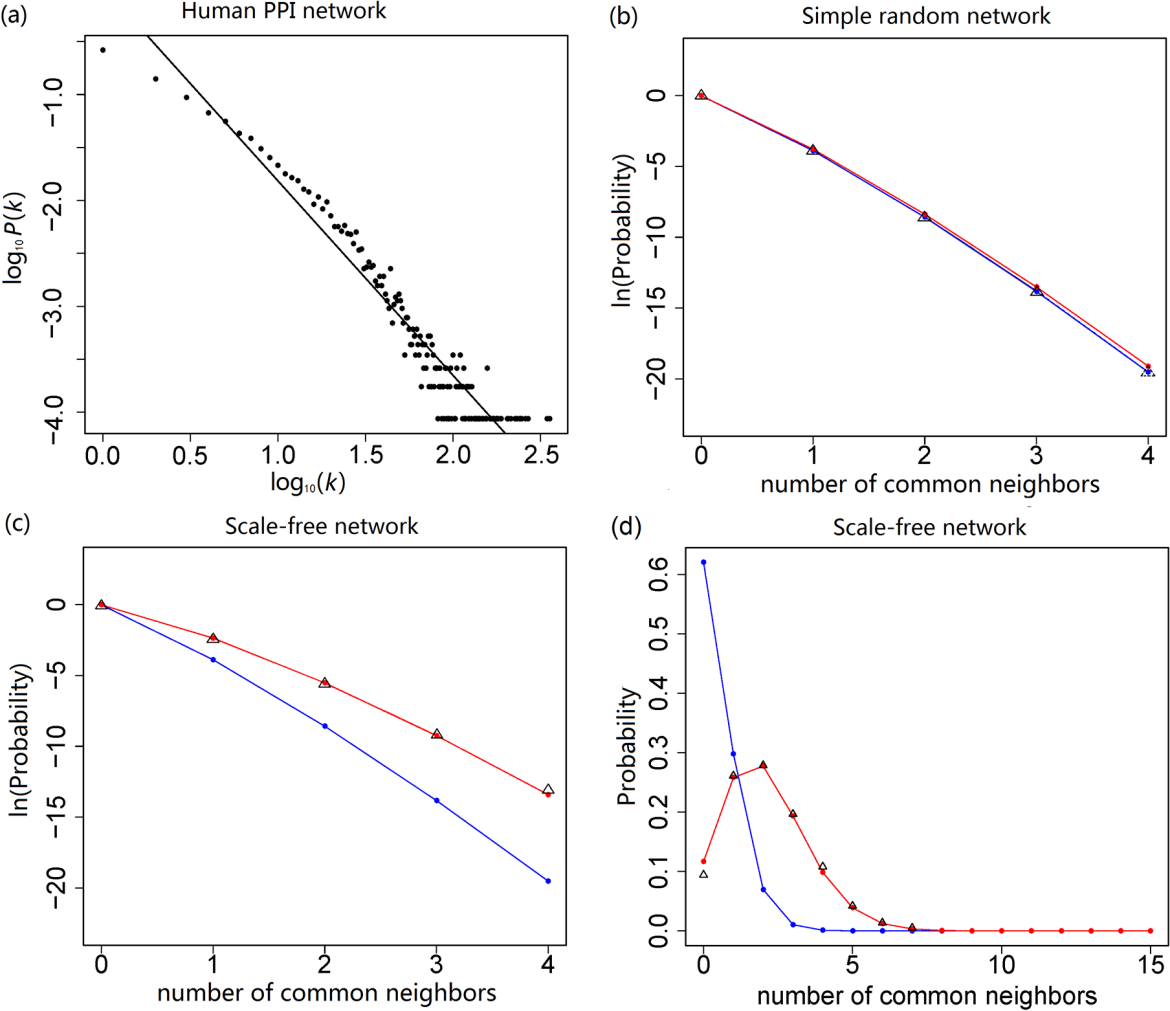
Comparison between the observed probabilities and the theoretical probabilities. (*a*) A human PPI network with *n* = 11,524 nodes and average degree of 9.0. The dashed line (fitted after log-log transformation) has a slope of -1.925 (the 95% confidence interval: [1.834, 2.016]) for the power-law tail (i.e., degree (*k*) ≥5). (*b*), (*c*) Performance comparison between formulas (1) and (2) in simple random networks (1000-time simulations) and scale-free networks (100-time simulations). The Black triangles represent the observed probabilities for the shared number of common neighbors, and the black dashed triangle represents the expected observation because *m* = 4 was not observed in (*b*). The red and blue points (lines) represent the theoretical probabilities calculated from formulas (1) and (2), respectively. Both (*b*) and (*c*) are examples with *k*_*A*_ = 15 and *k*_*B*_ = 16 as the degrees of protein *A* and *B*. (*d*) is also an example for scale-free networks, with *k*_*A*_ = 77 and *k*_*B*_ = 71 as the degrees of protein *A* and *B*, but without log-transformation of the probabilities (*y*-axis).

We performed Monte Carlo simulations to compare the probabilities from formulas (1) and (2). Our simulation was based on a human PPI network with 11,524 nodes and 51,840 links (see Materials and Methods). The degree distribution of the network followed a power-law distribution: *P*(*k*)∼*k* ^−*γ*^ (*k* is the degree; and *γ* is the degree exponent [18]), with *γ* equaling 1.925 for the power-law tail of *k* ≥ 5 (Fig 1 *A*). Thus, this PPI network is a scale-free network, which is in accordance with previous publications [3,17]. Based on this network, we used two methods to generate suitable random networks [15]. The method (*i*) is that, under the condition that all the nodes had an equal probability of being connected, we randomly added 51,840 links between 11,524 nodes. This method yielded simple random networks of which the degrees followed the Poisson distribution [22]. The method (*ii*) is that, based on the human PPI network, we randomly switched the neighbors of all nodes so that the degree of each node remained the same but the neighbors were randomly picked. This yielded (randomized) scale-free networks with the same degree distribution as our human PPI network (Fig 1 *A*). Method (*ii*) fulfilled our assumption on the PA property. By counting the number (*m*) of common neighbors for various combinations of *k*_*A*_ and *k*_*B*_ in networks generated by the two methods, we found that formula (1) yielded probabilities that almost matched the observations in simple random networks (Fig 1B) and nearly perfectly matched the observations in scale-free networks (Fig 1*C* and 1*D*). By contrast, although probabilities from formula (2) well matched the observations in simple random networks (Fig 1B), they differed significantly from the observations in scale-free networks: as *m* increased, the yielded probabilities (after log transformation) became much smaller than the observed probabilities (Fig 1 *C*). Therefore, formula (1) can be considered as a generalization of formula (2) that fits both simple random network and scale-free network.

### Incorporation of direct links into PAND

Since each link in a biological network (defined as a direct link) directly shows the functional association between two nodes, we incorporated this information into PAND by converting a direct link into *λ* common neighbors (*λ* ≥ 0):

$$P_{SI}=P_{S}(m+\lambda \;*\;I\;|\;n,k_{A}+\lambda \;*\;I,k_{B}+\lambda \;*\;I)$$ (3)

**Fig 2:**
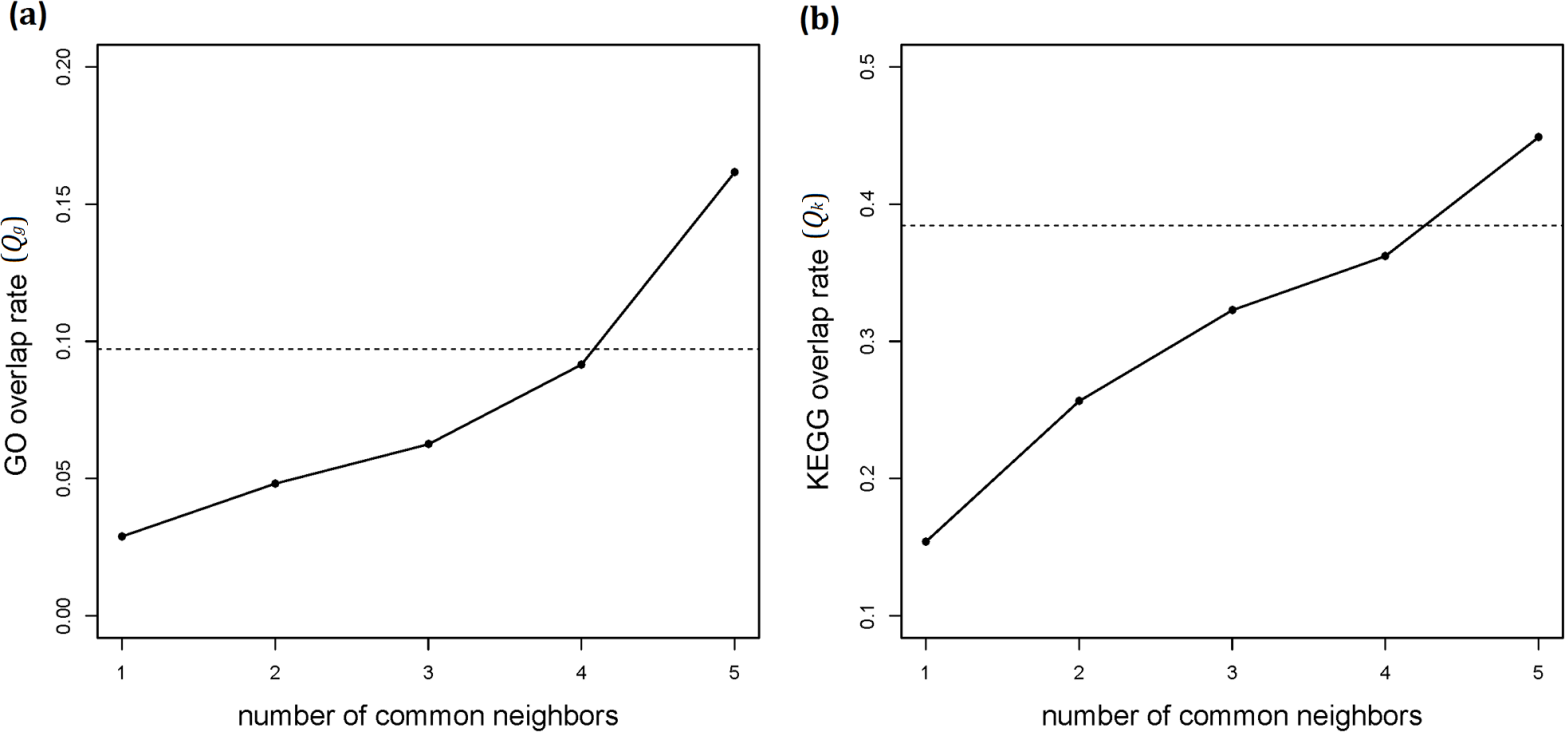
Comparison between direct interactions and indirect interactions. The *x*-axes are the number of common neighbors shared by proteins with only indirect interactions. The *y*-axes are the annotation overlap rates of GO (*a*) and KEGG (*b*). Dashed lines in both plots represent GO (*a*) and KEGG (*b*) annotation overlap rates for direct interactions. The annotation overlap rate (*Q*_*g*_ for GO and *Q*_*k*_ for KEGG) was used to assess the functional associations of protein pairs (see Materials and Methods for the definition).

Here *I* is a binary variable: *I* = 1 if there is a direct link between *A* and *B*; otherwise, *I* = 0. The integer *λ* (*λ* ≥ 0) is a weight we placed on the direct link and has different biological meanings with different values. *λ* = 0 indicates that a direct link gives no information on the functional association (thus *P*_*SI*_ is the same as *P*_*S*_); *λ* = 1 indicates that a direct link is as informative as sharing one common neighbor (defined as an indirect link) on the functional association; *λ* ≥ 2 indicates that a direct link is more informative than an indirect link. The effect of varying *λ* on *P*_*SI*_ is shown in S2 Fig. Since a direct link is usually derived from experiments, it represents a stronger evidence of the functional association than an indirect link. Specifically, in the real human PPI network, we proved this point by showing that protein pairs with only direct interactions (links) are more functionally associated than those with only indirect interactions of sharing less than five common neighbors (Fig 2). Therefore, *λ* should be greater than 1 to reflect this fact, and we arbitrarily chose *λ* = 2 in this study. We use “PAND” hereafter to refer to formula (3) with *λ* = 2, unless otherwise specified.

### Real data-based assessment of PAND

**Fig 3:**
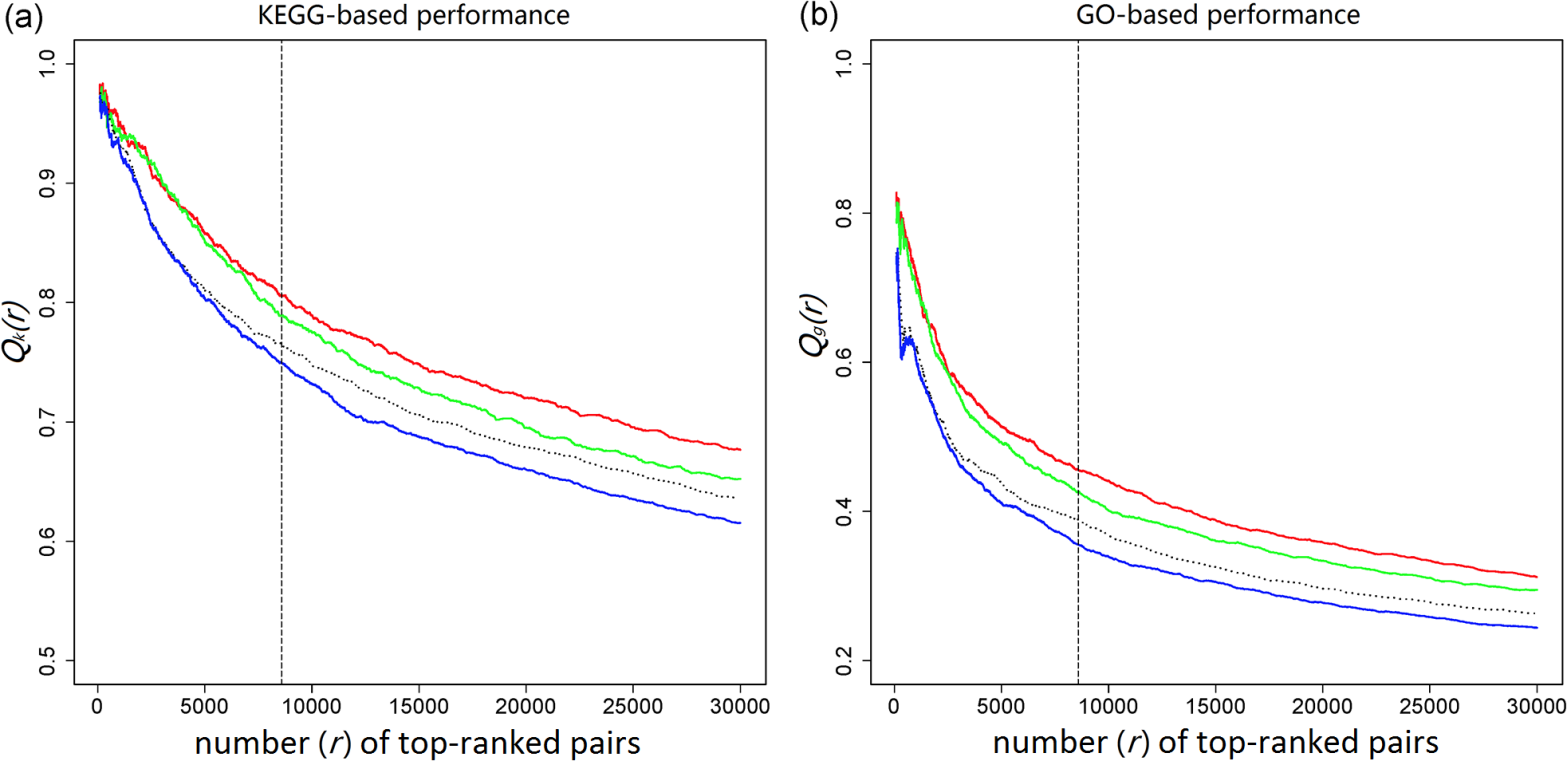
Comparison of the performance between *P*, *P*_*S*_ and *P*_*SI*_. In both plots, *x*-axes are the number (*r*) of top-ranked protein pairs (ranked by their probabilities: *P*, *P*_*S*_ and *P*_*SI*_); *y*-axes are the KEGG (*a*) and the GO (*b*) annotation overlap rates–***Q***_***k***_(***r***) and ***Q***_***g***_(***r***) for the top-ranked *r* protein pairs. Line colors represent the three formulas: green for (1), blue for (2) and red for (3). Dotted black lines (between green and blue lines) represent formula (2) with direct links integrated (with *λ* = 2). Vertical dashed lines (*r* = 8,583) represent the cut-off for significantly associated protein pairs. Fig.3 is based on the top-ranked 30,000 protein pairs from the three lists (each consists of over 1.5 million protein pairs).

As shown by Samanta and Liang (2003), those neighbor-sharing events with very small probabilities from formula (1) predicted functional associations between proteins in the PPI network of budding yeast. Here we applied formulas (1), (2) and (3) to the human PPI network and compared the quality of their derived top-ranked functional associations. Each protein pair with at least one common neighbor had three probabilities (i.e., *P*, *P*_*S*_ and *P*_*SI*_), which we used to rank the protein pairs in three different lists (i.e., each formula yielded one rank). We also used the corresponding p-values to rank the protein pairs and found that, in the human PPI network, the generated ranks were very similar to the above three ranked by *P*, *P*_*S*_ and *P*_*SI*_ (see Materials and Methods). As shown by Li and Liang (2009), a better formula would yield a list in which higher ranked protein pairs corresponded to better functional associations. We used GO and KEGG annotations as benchmarks to determine the functional association: if two proteins had any GO or KEGG annotation overlap, this protein pair was considered to be functionally associated. Based on this, we defined the GO annotation overlap rate (*Q*_*g*_) and the KEGG annotation overlap rate (*Q*_*k*_) to assess the functional associations of the top-ranked protein pairs (Materials and Methods). After comparing *Q*_*g*_ and *Q*_*k*_ between the three lists (Fig 3), we confirmed that, formula (1) yielded top-ranked protein pairs with better functional associations than formula (2), and formula (3) yielded top-ranked protein pairs with the best functional associations. Thus, for the same amount of top-ranked protein pairs, formula (3) yielded the best precision and recall rate in the human PPI network. (More comparison between *P*, *P*_*S*_ and *P*_*SI*_ can be found in S3 Fig) We also assessed the performance improvement of formulas (1) and (3) based on the top 30,000 protein pairs of Fig 3: compared with formula (2), formula (1) improved *Q*_*g*_ by 21% and *Q*_*k*_ by 6%; formula (3) further improved *Q*_*g*_ by 6% and *Q*_*k*_ by 4% when compared with formula (1). More importantly, even if direct links were incorporated into formula (2) in the same way as in formula (3) (with *λ* = 2), the subsequent *Q*_*g*_ and *Q*_*k*_ (dotted black curves in Fig 3) were still lower than that from formula (1), showing that the integration of PA assumption into formula (2) led to more performance improvement than simply integrating the information of direct links into formula (2). The above results show that, in scale-free networks, PAND-derived functional associations are more reliable than those from formula (2) that was developed without PA assumption [11].

### Comparison between the PPI network and the PAND-derived network

We further built three new networks with the top-ranked 51,840 functional links (associations) derived from each formula and calculated *Q*_*g*_ and *Q*_*k*_ for all the 51,840 links in each network (51,840 is the size of the human PPI network). For formula (3) (i.e., PAND), *Q*_*g*_ = 25% and *Q*_*k*_ = 61%; for formula (1), *Q*_*g*_ = 23% and *Q*_*k*_ = 58%; for formula (2), *Q*_*g*_ = 20% and *Q*_*k*_ = 56%. For the 51,840 links in the human PPI network, *Q*_*g*_ = 17% and *Q*_*k*_ = 51%, which were significantly lower than *Q*_*g*_ and *Q*_*k*_ for the PAND-derived network (p-value <10^−10^ by equal proportion test in R). This comparison demonstrated that the PAND-derived network had more reliable functional linkages than the human PPI network, thus should be a better source for further functional inference. In addition, only 13,454 (26%) links were common between the PAND-derived network and the human PPI network, showing that most of the PAND-derived links were new information not revealed by the PPI network itself.

To further evaluate the usefulness of the PAND-derived network, we applied the classical neighbor-counting approach proposed by Schwikowski *et al*. (2000) to the PAND-derived network and compared the results with those from the PPI network. The approach identified the most frequent function(s) among the direct neighbors of a protein and assigned the function(s) to the protein as the predicted functions [9]. Here we required the minimum frequency to be three and used the FDR (false discovery rate; see Materials and Methods) to assess the reliability of the predicted functions. Based on the PPI network, 2,334 KEGG annotations and 1,811 GO annotations were predicted with estimated FDRs of 41% and 78%, respectively. By contrast, with the PAND-derived network, 2,108 KEGG annotations and 1,658 GO annotations were predicted with estimated FDRs of 25% and 70%, respectively (the high FDR was attributed to the subset of GO terms used in this study; see Materials and Methods). The comparison between the FDRs showed that, with the same prediction approach, the PAND-derived network yielded higher-quality predictions, which supports the statement that the PAND-derived network is a better source for further functional inference.

### Functional inference based on a PAND-derived network

**Fig 4:**
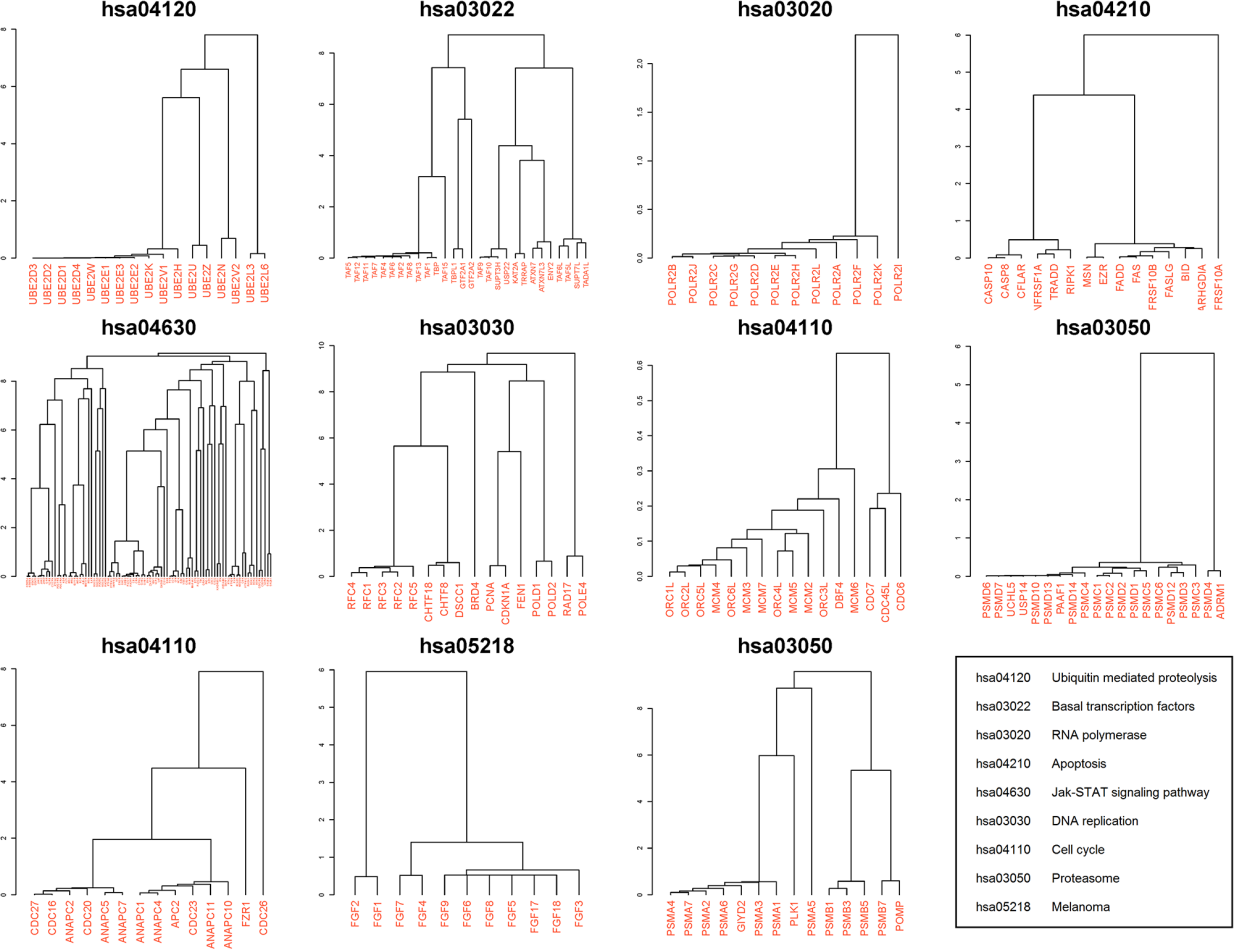
Subclusters significantly enriched in KEGG pathways with p-value < 10^−20^. The left bar in each plot shows the height of the subcluster in the whole cluster of 2,698 proteins. The name above each plot is the KEGG pathway ID corresponding to the most significant p-value. The bottom right panel maps the pathway IDs to the pathway names.

Since *P*_*SI*_ could be calculated for all (n2) possible combinations of node pairs in a network of size *n*, the cut-off for *P*_*SI*_ could be calculated in a way similar to the Bonferroni correction for p-values: Pcut=0.05(n2). Specifically, *P*_*cut*_ equals 7.53 × 10^−10^ for our human PPI network with *n* = 11,524. Using this stringent *P*_*cut*_, PAND yielded 8,583 significant protein pairs (i.e., protein pairs with *P*_*SI*_ < *P*_*cut*_; see S1 Table; biological meaning of these significant *P*_*SI*_ was discussed in Appendix A of S1 File and S4 Fig), of which strong functional associations have been observed (dashed lines in Fig 3). These protein pairs constituted a new network containing 2,796 nodes and 8,583 links. With this network, we first applied a direct annotation scheme (see Materials and Methods; [7]) and predicted 52 KEGG annotations for 52 proteins and 132 GO annotations for 132 proteins with estimated FDRs of 11% and 26%, respectively (see S5 Fig). By manual inspection (see Materials and Methods), we confirmed that ~46% of the predicted 184 annotations could be supported by existing evidence (see S2 Table), and we listed 34 predicted annotations in Table 1 that are worth further validation. We then applied a module-assisted scheme (see Materials and Methods; [7]) to cluster the nodes based on the *P*_*SI*_ of each link (see S6 Fig) and used a 3-step method (see Materials and Methods) to identify 11 informative subclusters (Fig 4). Each of the 11 subclusters was highly enriched in one KEGG pathway with p-value < 10^−20^, and we could further suggest possible KEGG annotations for these subcluster members (see S3 Table).

**Table 1:** Predicted GO and KEGG annotations that are worth further validation.

Protein	GO ID	GO term	p-value
JAK2	GO:0005159	insulin-like growth factor receptor binding	2.28E-12
PLCG1	GO:0005070	SH3/SH2 adaptor activity	1.23E-19
MSN	GO:0008633	activation of pro-apoptotic gene products	6.36E-17
MKRN3	GO:0051865	protein autoubiquitination	2.83E-11
DTX3L	GO:0051865	protein autoubiquitination	1.98E-11
UBOX5	GO:0051865	protein autoubiquitination	1.05E-11
RNF114	GO:0051865	protein autoubiquitination	1.36E-11
MID1	GO:0051865	protein autoubiquitination	1.36E-11
RASA1	GO:0042169	SH2 domain binding	1.39E-12
ARHGDIA	GO:0008633	activation of pro-apoptotic gene products	6.36E-17
RNF185	GO:0051865	protein autoubiquitination	2.24E-11
MAP2K7	GO:0005078	MAP-kinase scaffold activity	1.98E-11
IRS2	GO:0005159	insulin-like growth factor receptor binding	8.91E-11
UBE2U	GO:0070936	protein K48-linked ubiquitination	5.23E-23
KRT1	GO:0001533	cornified envelope	3.59E-11
PTPN1	GO:0005158	insulin receptor binding	6.41E-11
PI3	GO:0001533	cornified envelope	3.59E-11
GATAD2B	GO:0016581	NuRD complex	4.63E-11
Protein	**KEGG ID**	**KEGG pathway name**	**p-value**
PTPN6	hsa04664	Fc epsilon RI signaling pathway	1.13E-15
LYN	hsa04650	Natural killer cell mediated cytotoxicity	4.71E-15
MSN	hsa04210	Apoptosis	2.12E-14
LCP2	hsa04662	B cell receptor signaling pathway	6.36E-18
KIT	hsa05220	Chronic myeloid leukemia	1.90E-15
RASA1	hsa04650	Natural killer cell mediated cytotoxicity	4.09E-12
ARHGDIA	hsa04210	Apoptosis	2.12E-14
GIYD2	hsa03050	Proteasome	1.89E-16
INPP5D	hsa05220	Chronic myeloid leukemia	1.17E-11
GAB1	hsa05220	Chronic myeloid leukemia	5.25E-17
BLNK	hsa04664	Fc epsilon RI signaling pathway	1.41E-12
PAG1	hsa04650	Natural killer cell mediated cytotoxicity	4.18E-17
CHTF18	hsa03430	Mismatch repair	1.38E-15
PTK2B	hsa04012	ErbB signaling pathway	6.36E-13
PTPN1	hsa04722	Neurotrophin signaling pathway	6.94E-11
MAP4K1	hsa04664	Fc epsilon RI signaling pathway	2.58E-11

P-values were calculated by Fisher’s exact test based on the annotations of all significant partners for each protein. All these predictions are marked with “likely” in S2 Table. For more discussion on the prediction of GO and KEGG annotations, please refer to Appendix B of S1 File **.**

### Robustness analysis of PAND

Based on the human PPI network, we showed that PAND is robust: it is sensitive neither to a high false positive rate of PPI data, nor to a high error rate of gene annotations. After we added 25,920 false PPIs (50% of original PPIs) into our human PPI network, PAND still recovered ~87% of the 8,583 significant protein pairs within its own top-ranked 8,583 protein pairs. After we added 6,878 false GO annotations and 6,466 false KEGG annotations, PAND was still able to yield almost the same results on predicted annotations (~95% of predicted GO annotations and ~96% of predicted KEGG annotations were the same). Therefore, PAND is quite suitable for noisy data where the links and annotations suffer a high false positive rate.

### R package: PANDA

For easy implementation of the methods used above, we have provided an R package called *PANDA* (PAND-derived functional Associations). Given a biological network (in the format of binary interactions), *PANDA* will be able to perform the following tasks: (1) use PAND to calculate the *P*_*SI*_ (or p-value) for each pair of nodes and identify significantly associated nodes; (2) perform agglomerative hierarchical clustering based on the significantly associated nodes and generate a plot of the whole cluster; (3) predict GO terms and KEGG pathways for nodes; (4) identify subclusters whose members are enriched in KEGG pathways [(3) and (4) are performed only for PPI networks; fore more details, refer to S2 File (the Vignette)]. All functions in this package are implemented with the same methods as stated in the section of “Materials and Methods”. This PANDA package is provided as S3 File and has been deposited in CRAN (http://cran.r-project.org/) for future updates.

## Discussion

In this study, we developed an analytical method (PAND) to compute probabilities of common-neighbor sharing events and derived novel and reliable functional links between nodes within a large scale-free network. Our work has made at least two important contributions: first, formula (1) has proven to be an appropriate null distribution for accurately calculating probabilities of the neighbor-sharing events in biological networks with the PA probability linearly proportional to the node degree. Determining the probabilities of such events occurring in a random network requires high-resolution result where an analytical solution is preferred. This is because the probability we are interested in observing is typically on the order of 10^−10^, which is computing-intensive for Monte Carlo simulation methods where an impractical large number of sampling is required. Second, PAND is able to derive a new network with more reliable functional linkages than the human PPI network. This means the PAND-derived network is a better source for further functional inference. Based on this network, the FDR of functional predictions using existing annotation schemes can be improved. As shown in our example, both the direct and module-assisted approaches made high-quality functional predictions based on the PAND-derived network. Thus, PAND can also be considered as a valuable addition to the existing prediction schemes that are based on the links of scale-free networks.

Although PAND is based on the PA assumption that the connection probability is linearly proportional to the node degree (i.e., $P_{i}=\frac{k_{i}}{\sum_{l=1}^{n}k_{l}}$, see Materials and Methods), its application is not limited to the type of networks where this assumption holds. For example, PAND also gives nearly perfect estimation of the neighbor-sharing probabilities for the generated simple random networks [22], as long as the average degree is much smaller than the network size (so that *ϕ* will be close to 1). Since there is no PA property in simple random networks, the PA probability is the same for all nodes: $P_{i}=\frac{1}{n}=\frac{{k_{i}}^{0}}{\sum_{l=1}^{n}{k_{l}}^{0}}$. Thus, PANDA has excellent performance in networks with $P_{i}=\frac{k_{i}}{\sum_{l=1}^{n}k_{l}}$ or $P_{i}=\frac{{k_{i}}^{0}}{\sum_{l=1}^{n}{k_{l}}^{0}}$. Based on this, we speculate that PAND may also have a good performance in networks with PA probabilities between $\frac{{k_{i}}^{0}}{\sum_{l=1}^{n}{k_{l}}^{0}}$ and $\frac{k_{i}}{\sum_{l=1}^{n}k_{l}}$, such as $P_{i}=\frac{{\log(k}_{i}+1)}{\sum_{l=1}^{n}{\log(k}_{l}+1)}$ and $P_{i}=\frac{{k_{i}}^{0.5}}{\sum_{l=1}^{n}{k_{l}}^{0.5}}$. However, for networks with PA probabilities stronger than $\frac{k_{i}}{\sum_{l=1}^{n}k_{l}}$, such as $P_{i}=\frac{e^{k_{i}}}{\sum_{l=1}^{n}e^{k_{l}}}$, PAND may not perform well because nodes added to the network will always be connected to hub nodes, which makes sharing a large number of common neighbors much easier. A further study to access the performance of PAND in networks with various PA probabilities would be quite interesting.

As shown in the literature [3,15,17,23], hub nodes play a very important role in scale-free networks. Here we preliminarily assessed the influence of hub nodes on functional predictions in the human PPI network. A hub protein can be as powerful as a non-hub protein in predicting the function of its direct interacting neighbors (data not shown). Therefore, there is no need to distinguish hub proteins from others when predicting functions from direct neighbors, such as in Schwikowski *et al* (2000). For indirect neighbor-based functional inference, however, it becomes a different story. For a protein with *d* neighbors, there were (d2) combinations of any 2 neighbors, and we calculated the number (*T*_*d*_) of combinations that shared GO annotations. For all proteins of the same degree *d*, we defined a GO annotation overlap rate: Og(d)=∑k=dTd/∑k=d(d2). (*k* denotes the degree). We found that, as *d* increased, *O*_*g*_(*d*) generally became smaller (see S7 Fig). For KEGG annotation, we defined *O*_*k*_(*d*) in the same way and observed overlap rates similar to GO (see S7 Fig). As shown in S7 Fig, a hub protein becomes less potent for claiming the functional association of any two proteins that share this hub protein. In fact, for a common neighbor, there is a negative correlation between its degree and the predictive power it owns in the common neighbor-based functional predictions, which justifies the needs to reduce the influence of hub proteins. A pioneering research on this issue has been performed in Li and Liang (2009), but the proposed method of using two algorithms together is inconvenient to implement. Therefore, how to incorporate the information of hub proteins into PAND will certainly be an interesting part of our future work.

## Materials and Methods

### Derivation of formula (1)

Samanta and Liang (2003) developed a statistical model to calculate the probability of two nodes sharing a certain number of common neighbors in a PPI network. They showed that a very small probability corresponds to two nodes sharing more neighbors than expected by chance, which indicates a close functional relationship between the two nodes. Although the PPI network is a scale-free network, the scale-free property was not taken into account when their model was developed. Here we develop a new model as follows to calculate the probability of the same events in scale-free network due to its prevalence in biological networks. For a scale-free network of size *n*, we used Ω = {1,2, …, *n*} to denote all the nodes and used *k*_*i*_ to denote the degree of node *i* (*i* ∊ Ω). Suppose we add two new nodes here: *A* and *B*, with *k*_*A*_ as the degree of node *A*, *k*_*B*_ as the degree of node *B*, and *m* as the number of common neighbors. To make the model derivation simple, we make the following suppositions: (*i*) node *A* has three neighbors {*a*, *b*, *c*} and node *B* has four neighbors {*a*, *b*, *d*, *e*}, thus *m* = 2, *k*_*A*_ = 3 and *k*_*B*_ = 4 (*A*, *B* ∊ Ω); and (*ii*) the degrees of {*a*, *b*, *c*, *d*, *e*} are {*k*_*a*_, *k*_*b*_, *k*_*c*_, *k*_*d*_, *k*_*e*_} (*a*,*b*,*c*,*d*,*e* ∊ Ω and *a*≠*b*≠*c*≠*d*≠*e*). We also assume that the preferential attachment probability follows the Barabasi-Albert (BA) model [18] ⎼ $P_{i}=\frac{k_{i}}{\sum_{l=1}^{n}k_{l}}$, (*i*, *l* ∊ Ω). Based on this assumption, we can derive the following probabilities:

$$P_{abc}=\text{Pr}\left(A\;\text{picks}\;\left\{a,b,c\right\}\right)=\frac{k_{a}k_{b}k_{c}}{\sum_{i_{1}=1}^{n}\sum_{i_{2}=i_{1}+1}^{n}\sum_{i_{3}=i_{2}+1}^{n}k_{i_{1}}k_{i_{2}}k_{i_{3}}}$$ (4)

$$P_{abde}=\text{Pr}\left(B\;\text{picks}\;\left\{a,b,d,e\right\}\right)=\frac{k_{a}k_{b}k_{d}k_{e}}{\sum_{l_{1}=1}^{n}\sum_{l_{2}=l_{1}+1}^{n}\sum_{l_{3}=l_{2}+1}^{n}\sum_{l_{4}=l_{3}+1}^{n}k_{l_{1}}k_{l_{2}}k_{l_{3}}k_{l_{4}}}$$ (5)

The reason for the restriction on the summation indices in (4) and (5) is that we count each configuration only once. By further assuming that (4) and (5) are independent of each other, we have:

$$\text{Pr}(A\;\text{picks}\;\{a,b,c\}\&B\;\text{picks}\;\{a,b,d,e\})=P_{abc}\times P_{abde}$$

Here, the total number of unique ways of *A* and *B* sharing 2 common neighbors is (n2)(n−21)(n−32). The first term (n2) is the number of ways to choose node *a* and *b* from all *n* nodes; the second term is the number of ways to choose node *c* from the left *n*-2 nodes; the third term is the number of ways to choose node *d* and *e* from the left *n*-3 nodes. Therefore, the total probability of *A* and *B* sharing *m* = 2 nodes can be written as follows:

$$⇨\;Prob\left(m=2|k_{A},k_{B},n\right)=\sum_{a=1}^{n}\sum_{b=a+1}^{n}\sum_{c=1}^{n}\sum_{d=1}^{n}\sum_{e=d+1}^{n}P_{abc}\times P_{abde}$$ (6)

$$=\frac{\sum_{a=1}^{n}\sum_{b=a+1}^{n}\sum_{c=1}^{n}\sum_{d=1}^{n}\sum_{e=d+1}^{n}k_{a}^{2}k_{b}^{2}k_{c}k_{d}k_{e}}{\sum_{i_{1}=1}^{n}\sum_{i_{2}=i_{1}+1}^{n}\sum_{i_{3}=i_{2}+1}^{n}{k_{i_{1}}k}_{i_{2}}k_{i_{3}}\;\sum_{l_{1}=1}^{n}\sum_{l_{2}=l_{1}+1}^{n}\sum_{l_{3}=l_{2}+1}^{n}\sum_{l_{4}=l_{3}+1}^{n}{k_{l_{1}}k}_{l_{2}}k_{l_{3}}k_{l_{4}}}$$

The constraint (*a*≠*b*≠*c*≠*d*≠*e*) still exists in (6) although it is not shown for simplicity. Under the constraint, the total number of terms in the numerator is (n2)(n−21)(n−32). We further define S1, S2 and S as follows:

$$S=\sum_{a=1}^{n}\sum_{b=a+1}^{n}\sum_{c=1}^{n}\sum_{d=1}^{n}\sum_{e=d+1}^{n}k_{a}^{2}k_{b}^{2}k_{c}k_{d}k_{e}=\sum_{a=1}^{n}\sum_{b=a+1}^{n}k_{a}^{2}k_{b}^{2}\sum_{c=1}^{n}k_{c}\sum_{d=1}^{n}\sum_{e=d+1}^{n}k_{d}k_{e}$$

$$=\left(\begin{array}{c} n\\ 2 \end{array}\right)\;\bar{k_{a}^{2}k_{b}^{2}}\;\overset{∵c\neq a,b}{\overset{}{\overset{}{\overbrace{\left(\begin{array}{c} n-2\\ 1 \end{array}\right)}}}}\;\bar{k_{c}}\;\overset{∵d,e\neq a,b,c}{\overset{}{\overbrace{\overset{}{\left(\begin{array}{c} n-3\\ 2 \end{array}\right)}}}}\;\bar{k_{d}k_{e}}$$

(*a*≠*b*≠*c*≠*d*≠*e*)

$$S1=\sum_{i_{1}=1}^{n}\sum_{i_{2}=i_{1}+1}^{n}\sum_{i_{3}=i_{2}+1}^{n}k_{i_{1}}k_{i_{2}}k_{i_{3}}=\left(\begin{array}{c} n\\ 3 \end{array}\right)\bar{k_{i_{1}}k_{i_{2}}k_{i_{3}}}$$

(*i*_*1*_≠*i*_*2*_≠*i*_*3*_)

$$S2=\sum_{l_{1}=1}^{n}\sum_{l_{2}=l_{1}+1}^{n}\sum_{l_{3}=l_{2}+1}^{n}\sum_{l_{4}=l_{3}+1}^{n}k_{l_{1}}k_{l_{2}}k_{l_{3}}k_{l_{4}}=\left(\begin{array}{c} n\\ 4 \end{array}\right)\bar{k_{l_{1}}k_{l_{2}}k_{l_{3}}k_{l_{4}}}$$

(*l*_*1*_≠*l*_*2*_≠*l*_*3*_≠*l*_*4*_)

“$\bar{ABC}$” denotes the arithmetic mean. In human PPI networks, because *n* is always very large (typically, *n*≥10,000), we can have the following approximations by removing the constraints (*a*≠*b*≠*c*≠*d*≠*e*, *i*_*1*_≠*i*_*2*_≠*i*_*3*_ and *l*_*1*_≠*l*_*2*_≠*l*_*3*_≠*l*_*4*_) in S1, S2 and S:

S≈(n2)ka2¯kb2¯(n−21)kc¯(n−32)kd¯ke¯

=(n2)E(K2)E(K2)(n−21)E(K)(n−32)E(K)E(K)

=(n2)(n−21)(n−32)[E(K2)]2[E(K)]3

S1≈(n3)[E(K)]3,S2≈(n4)[E(K)]4

Here, *E*(*K*) is the arithmetic mean of the degrees of all nodes in the network.

$$⇨\;\text{Pr}\left(m=2\;|\;k_{A}=3,k_{B}=4,n\right)=\frac{S}{S1\times S2}=\frac{\left(\begin{array}{c} n\\ 2 \end{array}\right)\left(\begin{array}{c} n-2\\ 1 \end{array}\right)\left(\begin{array}{c} n-3\\ 2 \end{array}\right){\left[E\left(K^{2}\right)\right]}^{2}}{\left(\begin{array}{c} n\\ 3 \end{array}\right)\left(\begin{array}{c} n\\ 4 \end{array}\right){\left[E\left(K\right)\right]}^{2\times 2}}$$ (7)

More generally, the numerator (*S*) of Eq (6) can be derived as follows:

$$S=\overset{m}{\overbrace{\sum_{l_{1}=1}^{N}\sum_{l_{2}=l_{1}+1}^{N}\cdots \sum_{l_{m}=l_{m-1}+1}^{N}\;}}k_{l_{1}}^{2}k_{l_{2}}^{2}\cdots k_{l_{m}}^{2}\overset{n_{A}-m}{\overbrace{\sum_{j_{1}=1}^{N}\sum_{j_{2}=j_{1}+1}^{N}\cdots \sum_{j_{n_{A}-m}=j_{n_{A}-m-1}+1}^{N}\;}}k_{j_{1}}k_{j_{2}}\cdots k_{j_{n_{A}-m}}$$

$$\overset{n_{B}-m}{\overbrace{\sum_{i_{1}=1}^{N}\sum_{i_{2}=i_{1}+1}^{N}\cdots \sum_{i_{n_{B}-m}=i_{n_{B}-m-1}+1}^{N}\;}}k_{i_{1}}k_{i_{2}}\cdots k_{i_{n_{B}-m}}$$

≈(nm)[E(K2)]m(n−mkA−m)[E(K)]kA−m(n−kAkB−m)[E(K)]kB−m

The denominator (*D*) of Eq (6) is derived as follows:

D=S1×S2

$$=\sum_{j_{1}=1}^{n}\sum_{j_{2}=j_{1}+1}^{n}\cdots \sum_{j_{k_{A}}=j_{k_{A}-1}+1}^{n}k_{j_{1}}k_{j_{2}}\cdots k_{j_{k_{A}}}\sum_{i_{1}=1}^{n}\sum_{i_{2}=i_{1}+1}^{n}\cdots \sum_{i_{k_{B}}=i_{k_{B}-1}+1}^{n}k_{i_{1}}k_{i_{2}}\cdots k_{i_{k_{B}}}$$

≈(nkA)[E(K)]kA(nkB)[E(K)]kB

Therefore, in large scale-free networks:

$$P_{S0}\left(m|k_{A},k_{B},n\right)=\frac{S}{D}=\frac{\left(\begin{array}{c} n\\ m \end{array}\right){\left[E\left(K^{2}\right)\right]}^{m}\left(\begin{array}{c} n-m\\ k_{A}-m \end{array}\right){\left[E\left(K\right)\right]}^{k_{A}-m}\left(\begin{array}{c} n-k_{A}\\ k_{B}-m \end{array}\right){\left[E\left(K\right)\right]}^{k_{B}-m}}{\left(\begin{array}{c} n\\ k_{A} \end{array}\right){\left[E\left(K\right)\right]}^{k_{A}}\left(\begin{array}{c} n\\ k_{B} \end{array}\right){\left[E\left(K\right)\right]}^{k_{B}}}=\frac{\left(\begin{array}{c} n\\ m \end{array}\right)\left(\begin{array}{c} n-m\\ k_{A}-m \end{array}\right)\left(\begin{array}{c} n-k_{A}\\ k_{B}-m \end{array}\right){\left[E\left(K^{2}\right)\right]}^{m}}{\left(\begin{array}{c} n\\ k_{A} \end{array}\right)\left(\begin{array}{c} n\\ k_{B} \end{array}\right){\left[E\left(K\right)\right]}^{2m}}$$ (8)

Since there are some approximation steps, $\sum_{m=0}^{\underset{}{\min}\;\left(k_{A},\;k_{B}\right)}P_{S0}(m|k_{A},k_{B},n)$ is not equal to 1. Thus, a normalizing constant $\phi ={\left(\sum_{m=0}^{\underset{}{\min}\;\left(k_{A},\;k_{B}\right)}P_{S0}(m|k_{A},k_{B},n)\right)}^{-1}$ is needed so that $\sum_{m=0}^{\underset{}{\min}\;\left(k_{A},\;k_{B}\right)}\phi P_{S0}(m|k_{A},k_{B},n)=1$. Since *P*_*S*0_(*m*|*k*_*A*_,*k*_*B*_,*n*) is calculable for each *m*, *ϕ* is also calculable. Therefore, in large scale-free networks, we have:

$$P_{S}\left(m|k_{A},k_{B},n\right)=\phi P_{S0}\left(m|k_{A},k_{B},n\right)=\frac{\phi \left(\begin{array}{c} n\\ m \end{array}\right)\left(\begin{array}{c} n-m\\ k_{A}-m \end{array}\right)\left(\begin{array}{c} n-k_{A}\\ k_{B}-m \end{array}\right){\left[E\left(K^{2}\right)\right]}^{m}}{\left(\begin{array}{c} n\\ k_{A} \end{array}\right)\left(\begin{array}{c} n\\ k_{B} \end{array}\right){\left[E\left(K\right)\right]}^{2m}}$$ (1)

For this distribution, the one-tailed p-value is $\sum_{x=m}^{\underset{}{\min}\;\left(k_{A},\;k_{B}\right)}P_{S}(x|k_{A},k_{B},n)$.

In our human PPI network with network size *n* = 11,524, using one-tailed p-value to rank the associations between proteins yielded a result very similar to that by simply using *P*_*S*_ with *ϕ* = 1 (this led to ~2% difference for the top-ranked 10,000 associations; see S8 Fig), which was also true for *P*_*SI*_ (see S8 Fig) and *P* (see Ref [11]). This makes computation faster since only one probability for the observed *m* needs to be calculated to assess functional associations in the human PPI network, and we simply used *P*, *P*_*S*_ and *P*_*SI*_ to rank the functional association of each protein pair in this study (in our developed R package, there is an option to rank protein pairs by p-values).

### The human PPI network

We downloaded PPI data from two databases. We obtained 32,030 non-redundant PPIs for 9,445 unique proteins from the Biological General Repository for Interaction Datasets (BioGRID; Release 3.0.68; http://www.thebiogrid.org/) and 37,039 non-redundant PPIs for 9,465 unique proteins from the Human Protein Reference Database (HPRD; Release 9; http://www.hprd.org/). By combining these two databases, we obtained a PPI network with 51,840 non-redundant interactions between 11,524 proteins, of which < 900 are non-human proteins.

### Annotation databases

KEGG pathway annotations were downloaded from the KEGG website on August 21, 2009 (http://www.genome.jp/). The KEGG pathway maps proteins to the manually drawn pathways that represent the molecular interaction and reaction networks in various biological processes (such as metabolism and cellular processes) [21]. GO annotations were downloaded from the Gene Ontology website (submission data: 10/4/2010; http://www.geneontology.org/). The GO annotations (GO terms) map proteins to their associated biological processes, cellular components and molecular functions [20]. We used GO and KEGG pathway annotations to assess the functional associations between proteins and assign new annotations to proteins. To reduce the error rate of annotations, we removed GO annotations with evidence code “IEA” from the downloaded data. To improve the quality of functional inference, we only used the most specific GO terms (i.e., GO terms without any GO “offspring” terms) to perform GO-related analysis in this study.

We considered the KEGG annotation database to be independent of the PPI database because the two shared very few supporting literature (see Appendix C of S1 File). The GO annotation database shared a small fraction (~19%) of its supporting publications with the PPI database, but whether or not we removed the GO terms based on the overlapped publications from all analyses yielded the same conclusions as shown in above sections. As an example, we regenerated Fig 2a and Fig 3b in S9 Fig using only the GO annotations independent of the PPI database and reached the same conclusions.

### Definition of annotation overlap rate and FDR

Annotation overlap rate and FDR were calculated on the basis of the GO and KEGG databases described above. For *r* protein pairs, we defined their KEGG annotation overlap rate as follows: $Q_{k}(r)=\frac{T_{s}(r)}{T_{a}(r)}$. Here *k* denotes KEGG, *T*_*a*_(*r*) is the number of protein pairs of which both proteins have KEGG annotation, and *T*_s_(*r*) is the number of protein pairs that share at least one KEGG annotation. We defined the GO annotation overlap rate in the same way: $Q_{g}(r)=\frac{T_{s}(r)}{T_{a}(r)}$. For assigned GO or KEGG annotations, we defined FDR as follows: *FDR* = *Q*_*T*_/*Q*_*A*_. *Q*_*T*_ is the total number of falsely assigned annotations for proteins with known annotations (any assigned annotation that did not match the existing annotations was considered false); *Q*_*A*_ is the total number of assigned annotations for proteins with known annotations. Since GO and KEGG annotations may be far from complete, the FDRs were probably overestimated. As only the most specific GO terms were used in this study, the *Q*_*g*_ became relatively low and the GO-based FDR became relatively high compared with those from using more general GO terms (data not shown).

### Direct annotation scheme

We defined the partnership between two proteins to be significant if they were one of the 8,583 significant pairs. To assign new GO and KEGG annotations to a protein, we performed functional enrichment analysis (p-values were calculated by Fisher’s exact test) among a protein’s significant partners. We would assign an annotation if: (1) the p-value of this GO (or KEGG) annotation was the smallest among all enriched GO (or KEGG) annotations; and (2) the smallest p-value was also below a certain cut-off we had predetermined. By trying different cut-offs, we also estimated the corresponding FDRs (see the paragraph above) of the assigned annotations (see S5 Fig). To make our prediction more reliable, we picked 10^−10^ as the cut-off for the p-value, which yielded 52 KEGG annotations for 52 proteins and 132 GO annotations for 132 proteins with estimated FDRs of 11% and 26%, respectively (see S5 Fig). We have listed these predictions in S2 Table.

### Manual inspection for predicted GO and KEGG annotations

We used the following sources (in July 2011) to validate our predictions: (*i*) check the GO website to see if the human protein had any exact or more specific GO terms already assigned, (*ii*) check UniProt entry to see if there is curated information to support the predictions, (*iii*) check PubMed (i.e., read literature) to see if additional information can be obtained to support the predictions. For those unsupported predictions, if an assigned function could be reasonably inferred from existing literatures (or at least not contradictory to existing literatures), we marked them with “likely”; otherwise marked with “unlikely”. Examples of “supported”: “ligand-dependent nuclear receptor binding” for NCOA1 supported by an existing GO annotation, “SH3/SH2” for CBL supported by UniProtKB entry, and “SMAD protein signal transduction” for GDF5 supported by PMID: 20117381. An example of “likely”: “negative regulation of cholesterol storage” for RARA was inferred from PMID: 19886770. An example of “unlikely”: Med19 is a subunit of the mediator complex (PMID: 12584197), thus unlikely to be a part of RNA polymerase. However, it is known that mediator complex is involved in recruiting RNA polymerase [24], mediator complex co-localizes partially with RNA polymerase from ChIP-Seq assay [25]. Therefore in this case, the link between Med18 and RNA polymerase is biologically plausible.

### Module-assisted annotation scheme

After calculating the empirical cumulative distribution function (ECDF) from the *P*_*SI*_ of 8,127 significant protein pairs, we assigned each pair a score (between 0 and 1) from the ECDF in terms of its *P*_*SI*_. We then built a 2,698×2,698 dissimilarity matrix with the scores filled in as the distances between proteins. We further assigned “10” to the remaining (majority) blank slots of the matrix with the purpose of minimizing the background noise. With this matrix, we performed agglomerative hierarchical clustering, based on the unweighted group average. We showed a cluster of 2,698 members in S6 Fig. We proposed a 3-step method (see S10 Fig) as follows (taking pathway *Z* as an example here): Step 1: Pick a reasonable cut-off (height of the graph in S10 Fig) as a starting point to cut the whole cluster and identify base-level subclusters with members significantly enriched in *Z* (p-values were calculated by Fisher’s exact test); Step 2: Gradually move the cut-off towards a higher endpoint and calculate p-values iteratively on the subclusters that contain the identified base-level subclusters; Step 3: A subcluster with the most significant (smallest) p-value will be selected as the best subcluster for *Z*. Based on the structure of the whole cluster, we decided to use 1 as the starting point and 9.7 as the endpoint.

## Supporting Information

S1 Fig The flowchart that describes the important steps in this study and the logical relationship between them. The major conclusions of this study are also briefly described here.
(PNG)

S2 Fig The effect of varying *λ* on *P*_*SI*_. Different colors of points (lines) represent different *λ* (0, 1, 2, or 3) in formula (3), as described in the plot. In this example, *k*_*A*_ = 15 and *k*_*B*_ = 16 are the degrees of protein *A* and *B*. The *y*-axis (probability) has been log-transformed.
(TIF)

S3 Fig Distribution of the probabilities calculated by formulas (1), (2) and (3). We compared *P*, *P*_*S*_ and *P*_*SI*_ within 3 types of networks: (*a*) the human PPI network; (*b*) randomized scale-free networks; (*c*) simple random networks. [The generation of (*b*) and (*c*) was detailed in the section of simulation analysis of PAND]. The distributions of *P*_*S*_ and *P*_*SI*_ overlapped, making the curves yellow. These figures showed that, in scale-free networks (including the human PPI network), *P*_*S*_ and *P*_*SI*_ differed substantially from *P*; while in simple random networks, *P*_*S*_ and *P*_*SI*_ were almost identical to *P*.
(TIF)

S4 Fig GO-based evaluation of functional associations of significant pairs. BP: biological process; CC: cellular component; MF: molecular function. (*a*) GO annotation overlap rate (*Q*_*g*_) of significant pairs within each ontology. *T*_s_ is the number of protein pairs that share at least one GO term within the same GO ontology; *T*_*a*_ is the number of significant pairs that are both annotated within the same GO ontology. (*b*) Intersections between the 466 BP-shared, 674 CC-shared and 617 MF-shared protein pairs in (*a*). These results were obtained using the top 8,583 protein pairs.
(TIF)

S5 Fig FDR *vs*. p-value and FDR *vs*. number of predictions. (a), (b) The *x*-axes are the cut-offs of p-value below which we could assign annotations; the *y*-axes are the corresponding FDRs of those assigned annotations. (*c*), (*d*) The *x*-axes are the number of predictions of GO and KEGG annotations; the *y*-axes are the corresponding FDRs of the predictions. (a) and (c) are for GO, while (b) and (d) are for KEGG.
(TIF)

S6 Fig The cluster of 2,698 human proteins. The bar on the left side indicates the height in the cluster.
(PDF)

S7 Fig Common neighbors with larger degrees are less informative when predicting functional associations. For both plots, *x*-axes are the degrees of common neighbors; *y*-axes are corresponding annotation overlap rates for protein pairs that share the common neighbors with the degrees on the *x*-axes.
(TIF)

S8 Fig Comparison between using probabilities (*P*_*S*_ and *P*_*SI*_) and using p-values on ranking protein pairs. The protein pairs are ranked either by their probabilities or by their p-values yielded by formulas (1) or (3). The *y*-axis stands for the proportion of protein pairs shared by two groups of top-ranked protein pairs (*x*-axis)–one ranked by the probability and the other by the p-value yielded by the same formula. The red solid line compares the top-ranked protein pairs ranked by *P*_*SI*_ and the p-value yielded by formula (3), and the green dashed line compares *P*_*S*_ and the p-value yielded by formula (1). The vertical solid black line (*x* = 8,583) stands for the cut-off for significantly associated protein pairs, which corresponds to ~98% protein-pair overlap rate for both red and green lines.
(TIF)

S9 Fig Re-plotting Fig 3B and Fig 2A based on the GO annotations independent of the human PPI data. (*a*) Comparison of the performance between *P*, *P*_*S*_ and *P*_*SI*_. (*b*) Comparison between direct interactions and indirect interactions. The methods for plotting (*a*) and (*b*) (including the figure notations) are the same as for Fig 3B and Fig 2A, respectively.
(TIF)

S10 Fig The 3-step method to find informative subclusters. We first cut the cluster at a starting point (height of 1), then gradually moved the cut-off to higher levels with an interval of 0.1, toward an endpoint at the height of 9.7. With each cut-off, we performed enrichment analysis of each subcluster and compared them with those obtained from previous cut-offs.
(TIF)

S1 File Supporting Text. This text includes three parts: Appendix A: “Analysis of GO-term predictions”, Appendix B: “Possible biological meanings of the significant *P*_*SI*_ derived from PAND”, and Appendix C: “Analysis on the independence between the PPI dataset and the annotation datasets (GO and KEGG).
(PDF)

S2 File The Vignette for *PANDA*. (PDF)

S3 File The R package *PANDA*. (GZ)

S1 Table The 8,583 significant protein pairs derived by PAND. In this table, Column 3 is the natural log-transformed probability [i.e., log(*P*_*SI*_)] from PAND; Column 4 is the number of common neighbors of Column 1 and 2; Column 5 indicates if Column 1 and 2 have a direct interaction (1- yes, 0-no).
(TXT)

S2 Table Predictions of GO and KEGG pathway annotations. For each protein, the ratio shows the number of significant partners (denominator) and the number of significant partners with the assigned GO/KEGG annotation (numerator). P-values were calculated by Fisher’s exact test based on the annotations of all significant partners for each protein.
(DOCX)

S3 Table Functional inferences based on our clustering scheme. Each row corresponds to a subcluster in Fig 3 with the same KEGG ID. The 1^st^ column (Protein) lists the proteins without the KEGG annotation in the 2^nd^ column. Ratio equals the percentage of proteins with the same KEGG annotation within the subcluster; height equals the level at which the subcluster was obtained. P-values were calculated with Fisher’s exact test for each subcluster.
(DOCX)

## References

[1] Wood V, Harris MA, McDowall MD, Rutherford K, Vaughan BW, Staines DM, et al PomBase: a comprehensive online resource for fission yeast. Nucleic Acids Res 2012, 40: D695–D699. 22039153 10.1093/nar/gkr853 PMC3245111

[2] Vazquez A, Flammini A, Maritan A, Vespignani A. Global protein function prediction from protein-protein interaction networks. Nat Biotechnol 2003, 20: 697–700. 10.1038/nbt825 12740586

[3] Barabasi A-L, Oltvai ZN. Network biology: understanding the cell’s functional organization. Nature Rev Genet 2004, 5: 101–113. 14735121 10.1038/nrg1272

[4] Karaoz U, Murali TM, Letovsky S, Zheng Y, Ding C, Cantor CR, et al Whole-genome annotation by using evidence integration in functional-linkage networks. Proc Natl Acad Sci U S A 2004, 101: 2888–2893. 14981259 10.1073/pnas.0307326101 PMC365715

[5] Rual J, Venkatesan K, Hao T, Hirozane-Kishikawa T, Dricot A, Li N, et al Towards a proteome-scale map of the human protein–protein interaction network. Nature 2005, 437: 1173–1178. 16189514 10.1038/nature04209

[6] Albert R. Network inference, analysis, and modeling in systems biology. Plant Cell 2007, 19: 3327–3338. 18055607 10.1105/tpc.107.054700 PMC2174897

[7] Sharan R, Ulitsky I, Shamir R. Network-based prediction of protein function. Mol Syst Bio 2007, 3:88. 17353930 10.1038/msb4100129 PMC1847944

[8] Llewellyn R, Eisenberg DS. Annotating proteins with generalized functional linkages. Proc Natl Acad Sci U S A 2008, 105: 17700–17705. 19004787 10.1073/pnas.0809583105 PMC2584710

[9] Schwikowski B, Uetz P, Fields S. A network of protein–protein interactions in yeast. Nat Biotechnol 2000, 18: 1257–1261. 11101803 10.1038/82360

[10] Hishigaki H, Nakai K, Ono T, Tanigami A, Takagi T. Assessment of prediction accuracy of protein function from protein-protein interaction data. Yeast 2001, 18: 523–531. 11284008 10.1002/yea.706

[11] Samanta MP, Liang S. Predicting protein functions from redundancies in large-scale protein interaction networks. Proc Natl Acad Sci U S A 2003, 100: 12579–12583. 14566057 10.1073/pnas.2132527100 PMC240660

[12] Brun C, Chevenet F, Martin D, Wojcik J, Guénoche A, Jacq B. Functional classification of proteins for the prediction of cellular function from a protein-protein interaction network. Genome Biol 2003, 5: R6. 14709178 10.1186/gb-2003-5-1-r6 PMC395738

[13] Letovsky S, Kasif S. Predicting protein function from protein/protein interaction data: a probabilistic approach. Bioinformatics 2003, 19 Suppl. 1: i197–i204. 12855458 10.1093/bioinformatics/btg1026

[14] Chua HN, Sung WK, Wong L. Exploiting indirect neighbours and topological weight to predict protein function from protein-protein interactions. Bioinformatics 2006, 22: 1623–1630. 16632496 10.1093/bioinformatics/btl145

[15] Li H, Liang S. Local network topology in human protein interaction data predicts functional association. PLoS ONE 2009, 4(7): e6410. 19641626 10.1371/journal.pone.0006410 PMC2713831

[16] Nabieva E, Jim K, Agarwal A, Chazelle B, Singh M. Whole-proteome prediction of protein function via graph-theoretic analysis of interaction maps. Bioinformatics 2005, 21: i302–i310. 15961472 10.1093/bioinformatics/bti1054

[17] Barabasi AL. Scale-free networks: a decade and beyond. Science 2009, 325: 412–413. 19628854 10.1126/science.1173299

[18] Barabasi AL, Albert R. Emergence of scaling in random networks. Science 1999, 286: 509–512. 10521342 10.1126/science.286.5439.509

[19] Boccaletti S, Latora V, Moreno Y, Chavez M, Hwang D-U. Complex networks: structure and dynamics. Phys Rep 2006, 424: 175–308.

[20] Ashburner M, Ball CA, Blake JA, Botstein D, Butler H, Cherry JM, et al Gene ontology: tool for the unification of biology. Nat Genet 2000, 25: 25–29. 10802651 10.1038/75556 PMC3037419

[21] Kanehisa M, Goto S. KEGG: Kyoto Encyclopedia of Genes and Genomes. Nucleic Acids Res 2000, 28: 27–30. 10592173 10.1093/nar/28.1.27 PMC102409

[22] Erdos P, Renyi A. On random graphs. Publ Math (Debrecen) 1959, 6: 290.

[23] Vidal M, Cusick ME, Barabasi AL. Interactome Networks and Human Disease. Cell 2011, 144: 986–998. 21414488 10.1016/j.cell.2011.02.016 PMC3102045

[24] Kornberg RD. Mediator and the mechanism of transcriptional activation. Trends Biochem Sci 2005, 30(5):235–239. 15896740 10.1016/j.tibs.2005.03.011

[25] Kagey MH, Newman JJ, Bilodeau S, Zhan Y, Orlando DA, van Berkum NL, et al Mediator and cohesin connect gene expression and chromatin architecture. Nature 2010, 467(7314):430–435. 20720539 10.1038/nature09380 PMC2953795

