# Supplementary figures and images for: PAND: A Distribution to Identify Functional Linkage from Networks with Preferential Attachment Property
Source: PLoS One. 2015 Jul 9;10(7):e0127968. doi: 10.1371/journal.pone.0127968 (PMC4497646; doi:10.1371/journal.pone.0127968)

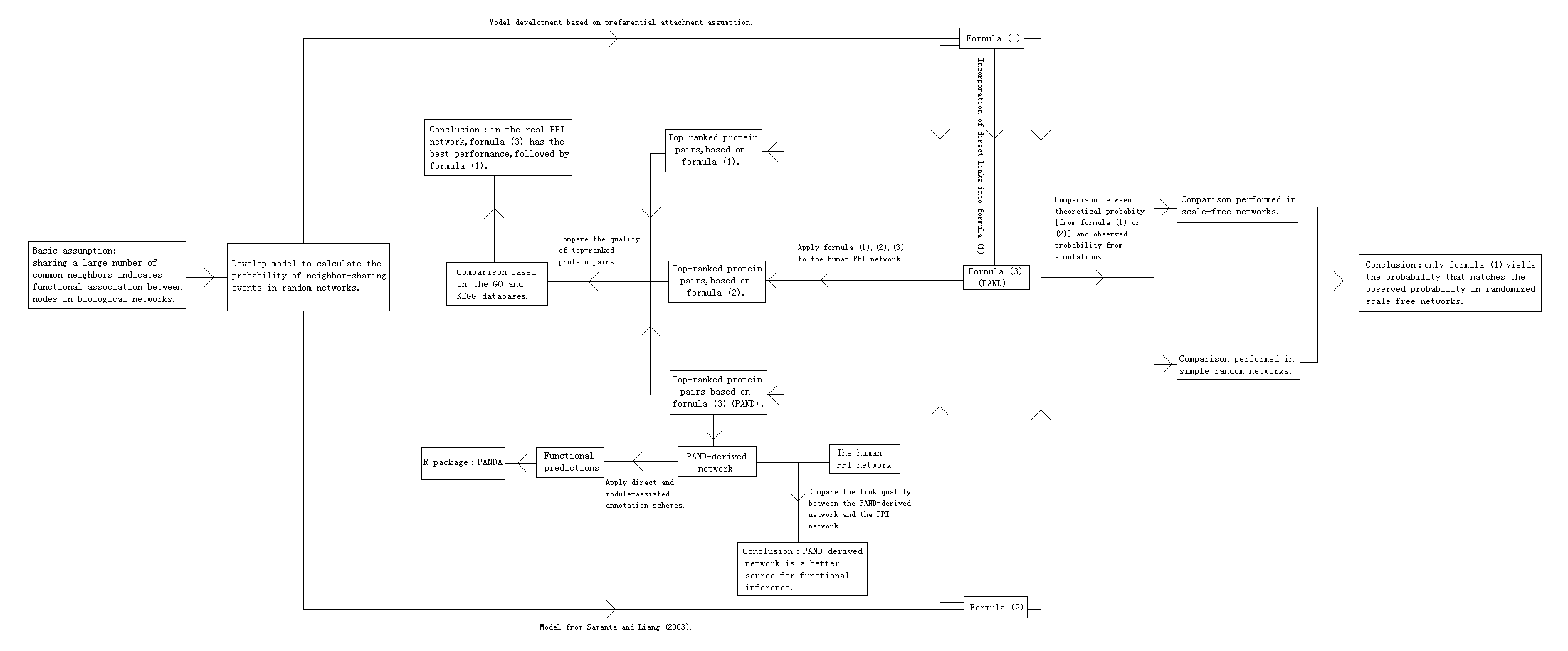

Supplement: S1 Fig — The major conclusions of this study are also briefly described here. (PNG) [file pone.0127968.s001.png]

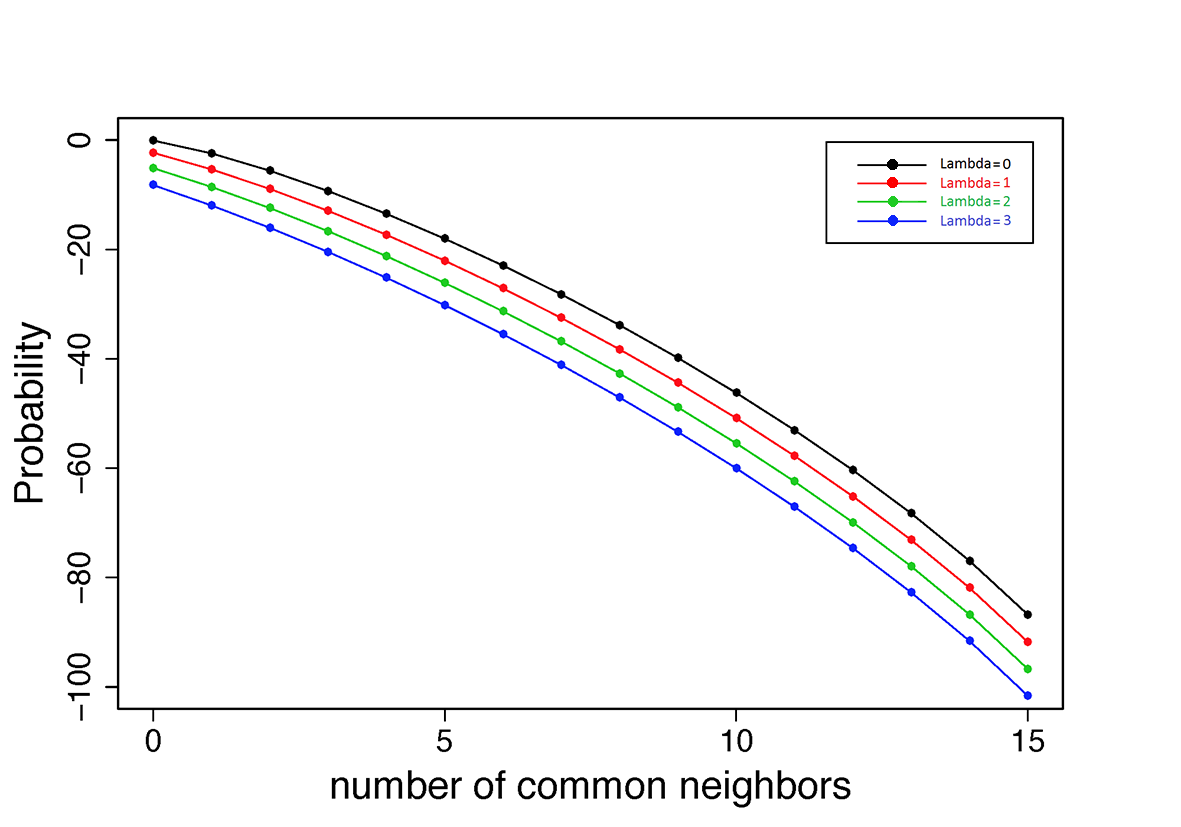

Supplement: S2 Fig — Different colors of points (lines) represent different λ (0, 1, 2, or 3) in formula (3), as described in the plot. In this example, k A = 15 and k B = 16 are the degrees of protein A and B. The y-axis (probability) has been log-transformed. (TIF) [file pone.0127968.s002.tif]

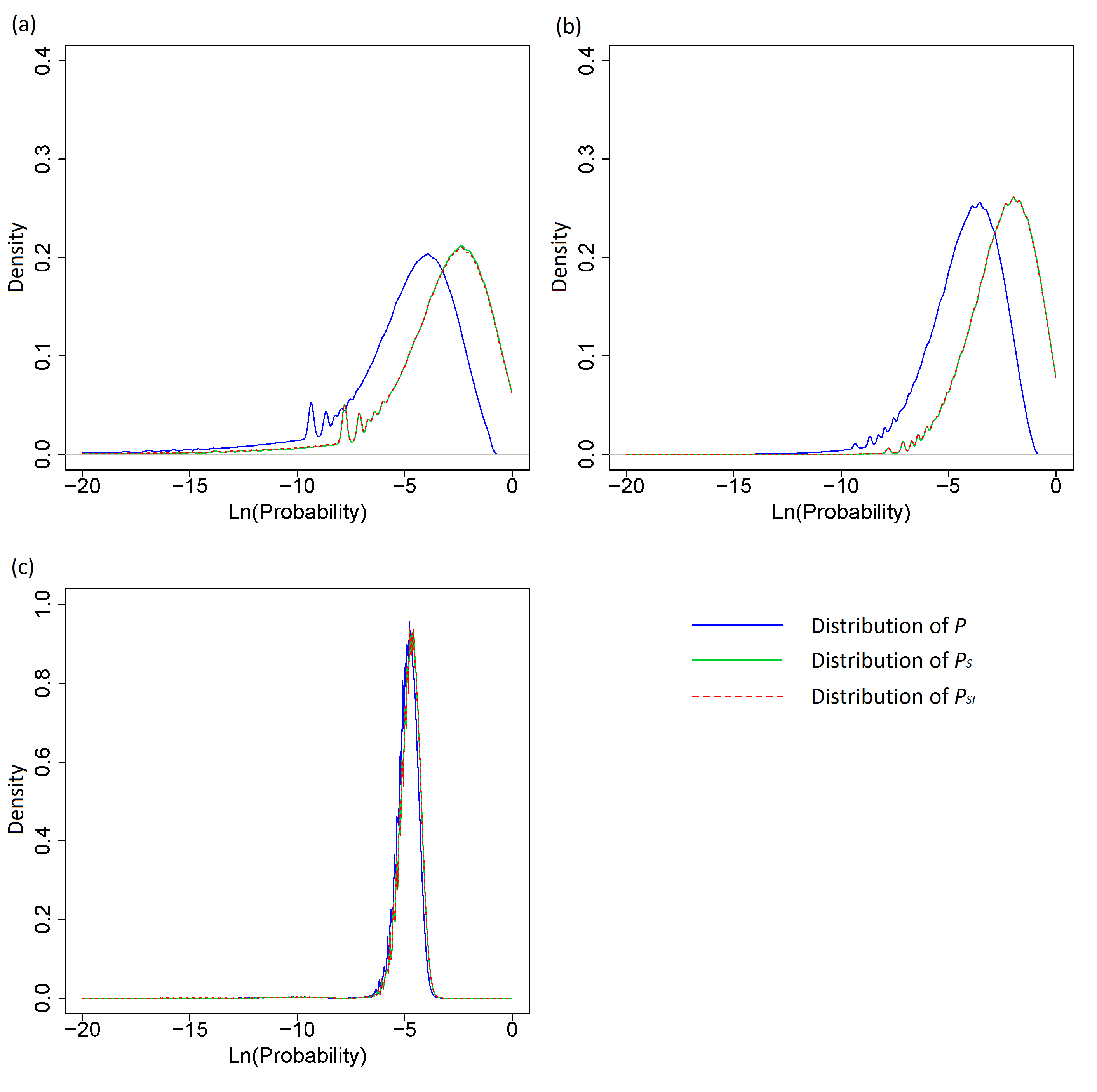

Supplement: S3 Fig — We compared P, P S and P SI within 3 types of networks: (a) the human PPI network; (b) randomized scale-free networks; (c) simple random networks. [The generation of (b) and (c) was detailed in the section of simulation analysis of PAND]. The distributions of P S and P SI overlapped, making the curves yellow. These figures showed that, in scale-free networks (including the human PPI network), P S and P SI differed substantially from P; while in simple random networks, P S and P SI were almost identical to P. (TIF) [file pone.0127968.s003.tif]

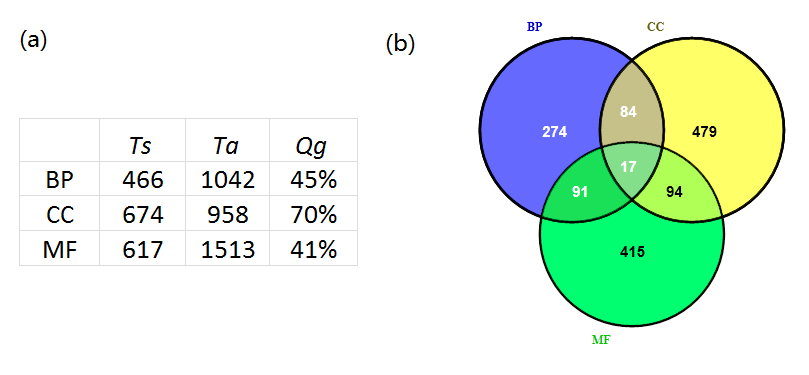

Supplement: S4 Fig — BP: biological process; CC: cellular component; MF: molecular function. (a) GO annotation overlap rate (Q g) of significant pairs within each ontology. T s is the number of protein pairs that share at least one GO term within the same GO ontology; T a is the number of significant pairs that are both annotated within the same GO ontology. (b) Intersections between the 466 BP-shared, 674 CC-shared and 617 MF-shared protein pairs in (a). These results were obtained using the top 8,583 protein pairs. (TIF) [file pone.0127968.s004.tif]

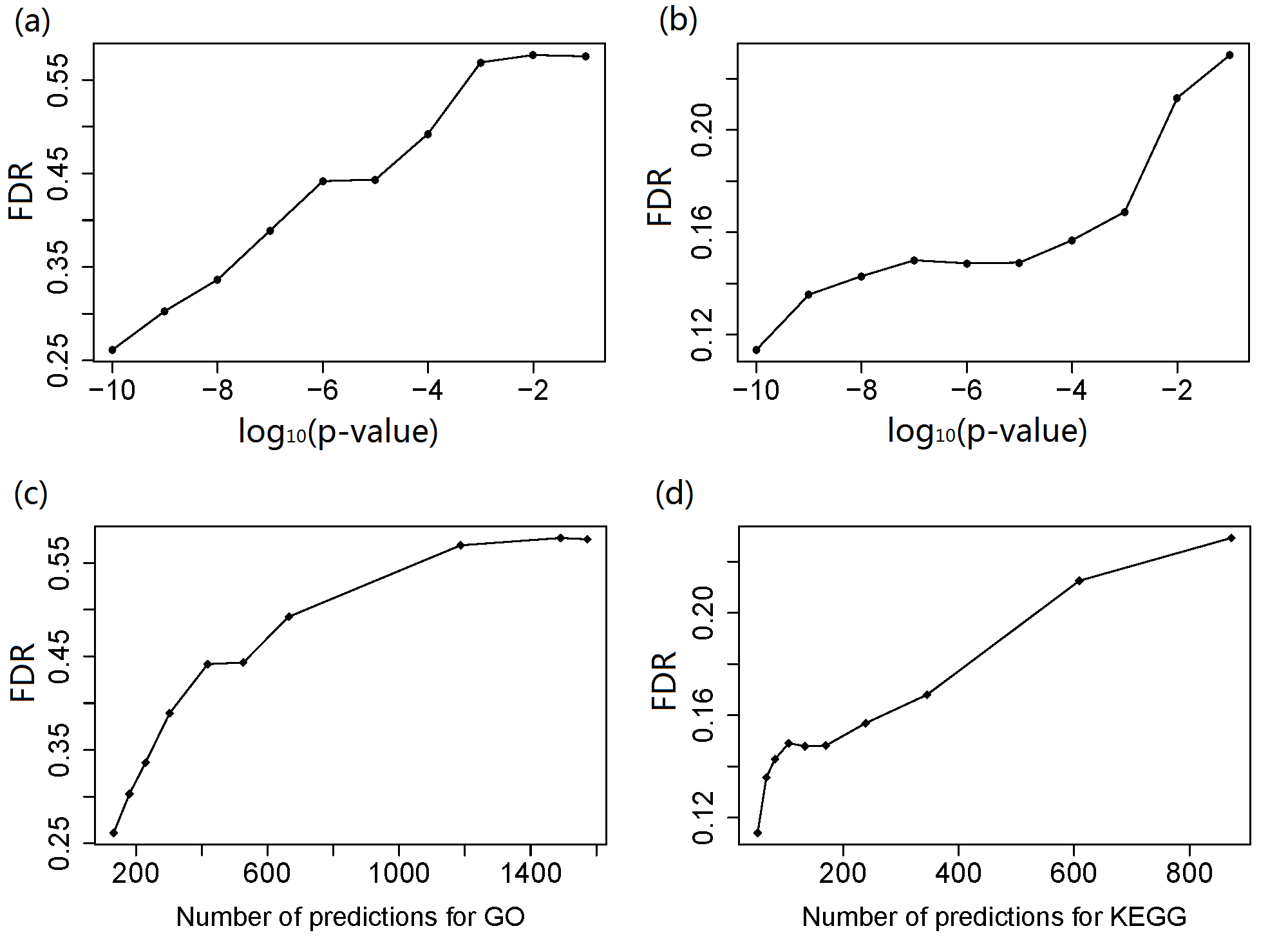

Supplement: S5 Fig — (a), (b) The x-axes are the cut-offs of p-value below which we could assign annotations; the y-axes are the corresponding FDRs of those assigned annotations. (c), (d) The x-axes are the number of predictions of GO and KEGG annotations; the y-axes are the corresponding FDRs of the predictions. (a) and (c) are for GO, while (b) and (d) are for KEGG. (TIF) [file pone.0127968.s005.tif]

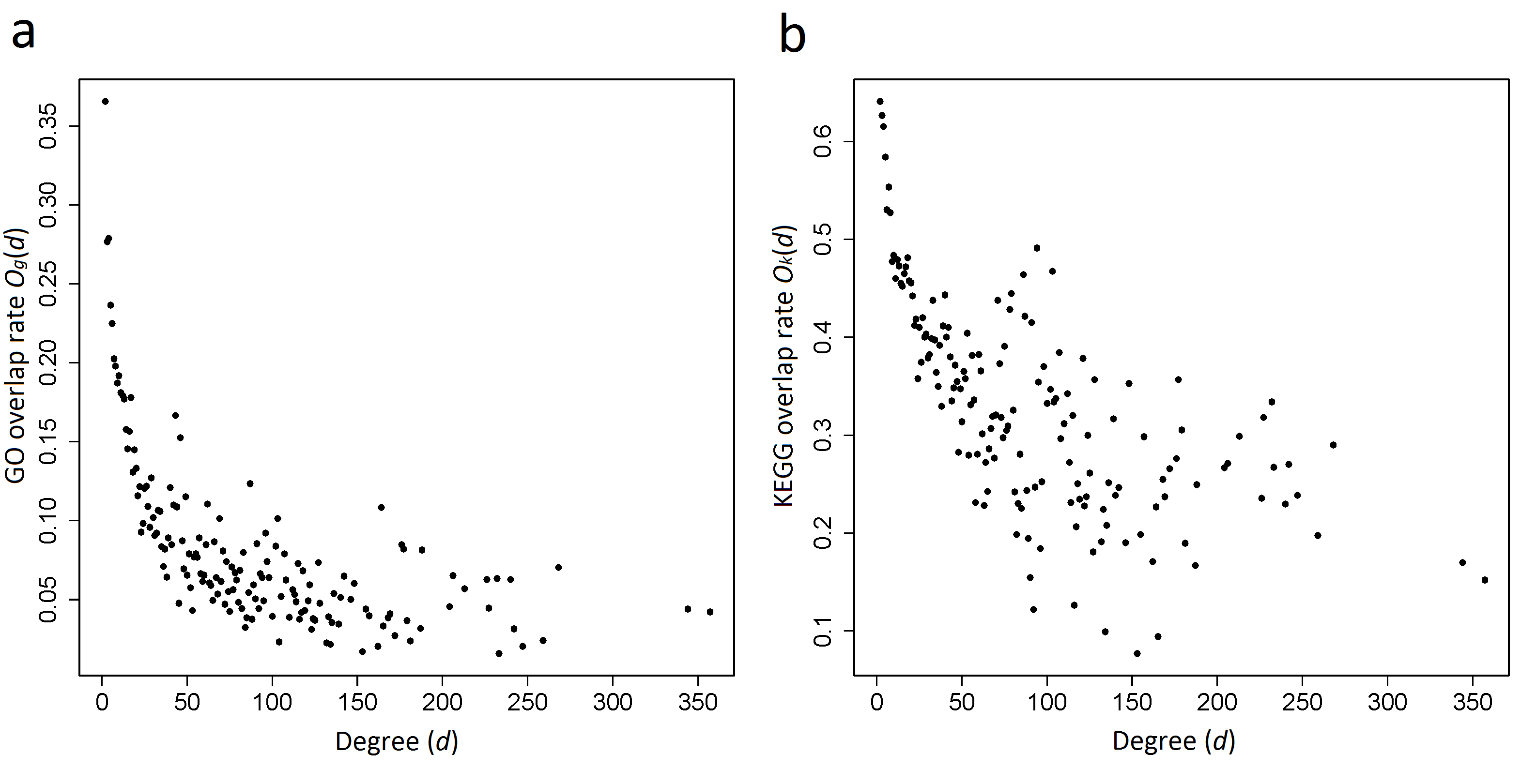

Supplement: S7 Fig — For both plots, x-axes are the degrees of common neighbors; y-axes are corresponding annotation overlap rates for protein pairs that share the common neighbors with the degrees on the x-axes. (TIF) [file pone.0127968.s007.tif]

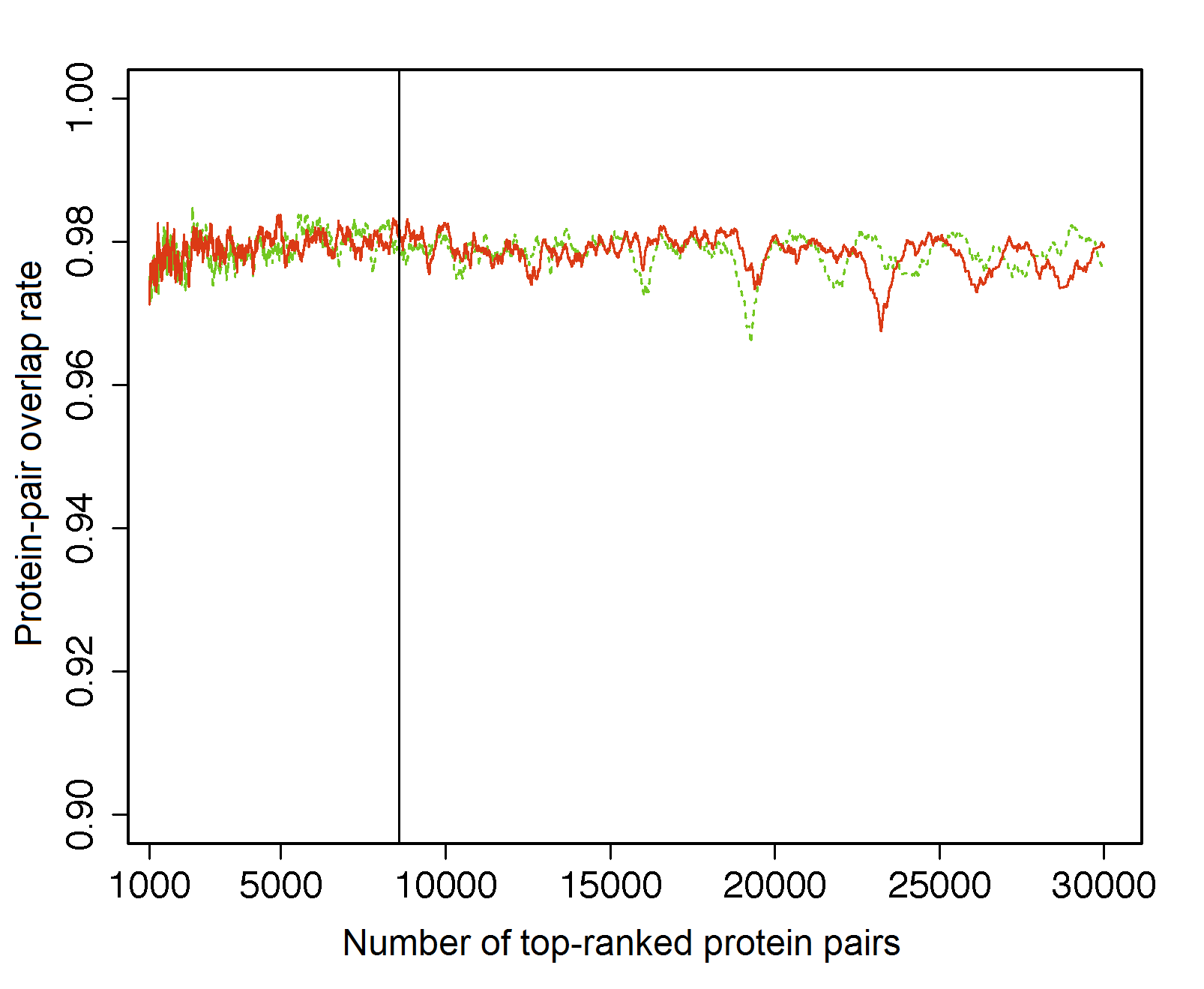

Supplement: S8 Fig — The protein pairs are ranked either by their probabilities or by their p-values yielded by formulas (1) or (3). The y-axis stands for the proportion of protein pairs shared by two groups of top-ranked protein pairs (x-axis)–one ranked by the probability and the other by the p-value yielded by the same formula. The red solid line compares the top-ranked protein pairs ranked by P SI and the p-value yielded by formula (3), and the green dashed line compares P S and the p-value yielded by formula (1). The vertical solid black line (x = 8,583) stands for the cut-off for significantly associated protein pairs, which corresponds to ~98% protein-pair overlap rate for both red and green lines. (TIF) [file pone.0127968.s008.tif]

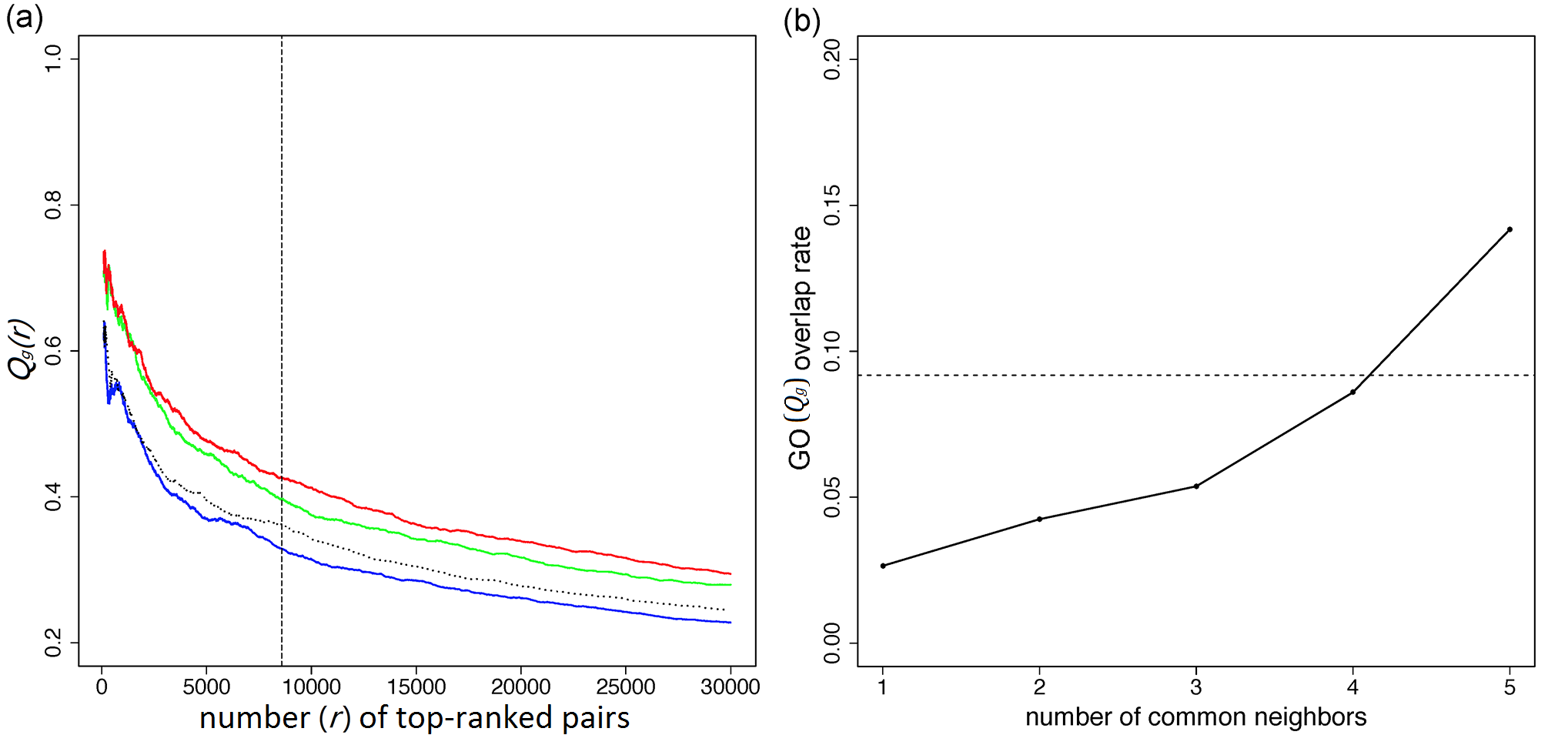

Supplement: S9 Fig — (a) Comparison of the performance between P, P S and P SI. (b) Comparison between direct interactions and indirect interactions. The methods for plotting (a) and (b) (including the figure notations) are the same as for Fig 3B and Fig 2A, respectively. (TIF) [file pone.0127968.s009.tif]

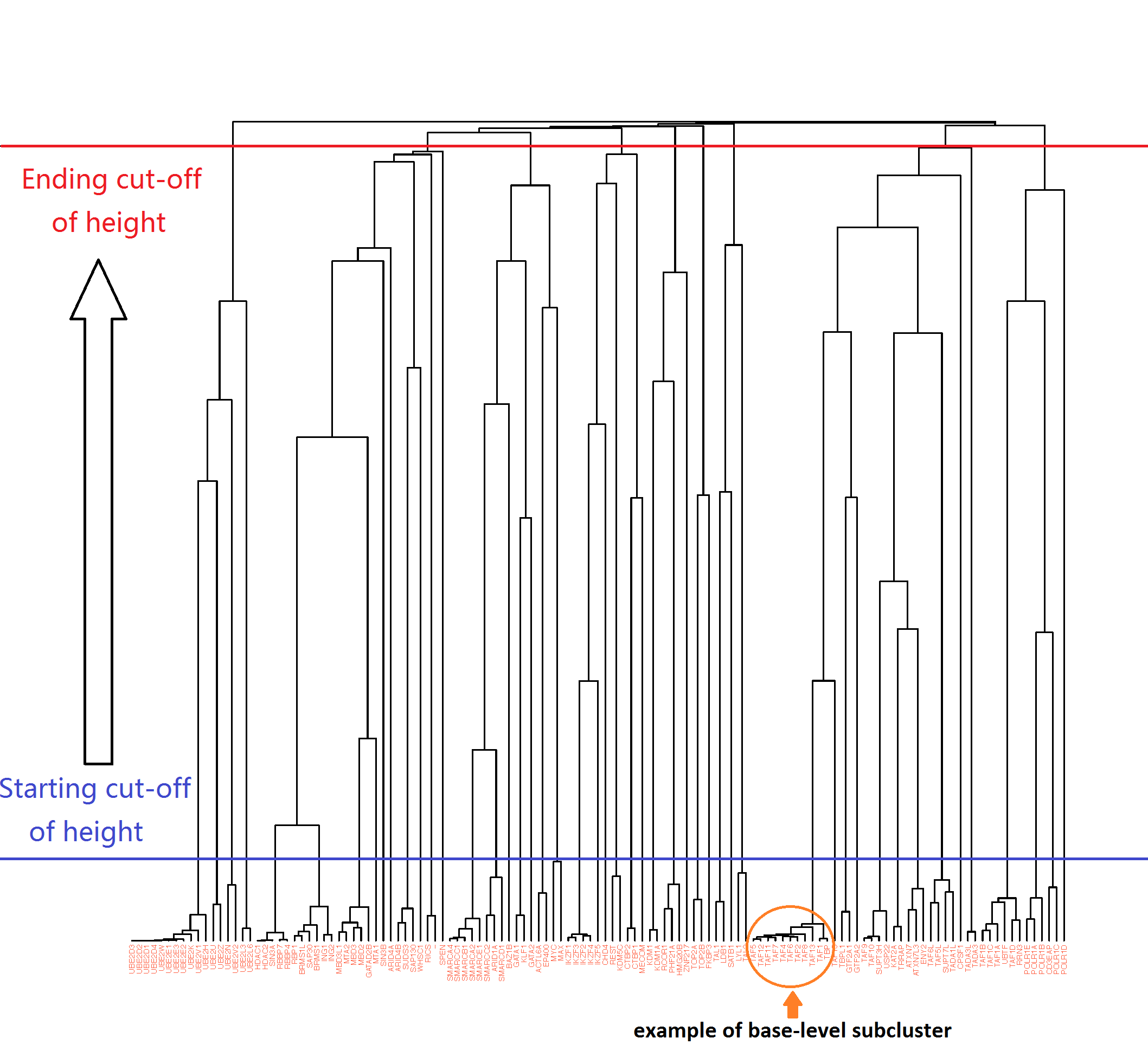

Supplement: S10 Fig — We first cut the cluster at a starting point (height of 1), then gradually moved the cut-off to higher levels with an interval of 0.1, toward an endpoint at the height of 9.7. With each cut-off, we performed enrichment analysis of each subcluster and compared them with those obtained from previous cut-offs. (TIF) [file pone.0127968.s010.tif]
