# Supplementary material for: PAND: A Distribution to Identify Functional Linkage from Networks with Preferential Attachment Property
Source: PLoS One. 2015 Jul 9;10(7):e0127968. doi: 10.1371/journal.pone.0127968 (PMC4497646; doi:10.1371/journal.pone.0127968)
Supplement: S1 File — This text includes three parts: Appendix A: “Analysis of GO-term predictions”, Appendix B: “Possible biological meanings of the significant P SI derived from PAND”, and Appendix C: “Analysis on the independence between the PPI dataset and the annotation datasets (GO and KEGG). (PDF) [file pone.0127968.s011.pdf]

PAND: a distribution to identify functional linkage from networks with preferential attachment property

Supporting Text

Contents

Appendix A: Possible biological meanings of the significant P_{SI} derived from PAND 3

Appendix B: Analysis of GO-term predictions 4

Appendix C: Analysis on the independence between the PPI dataset and the annotation datasets (GO and KEGG) 5

Appendix A: Possible biological meanings of the significant P_{SI} derived from PAND

To better understand the biological meaning of PAND-derived significant P_{SI} for PPI networks, we used the GO database to evaluate the associations of significant protein pairs (see S4 Fig.). For the 8,583 pairs, the annotation overlap rates were 70%, 45% and 41% for the cellular component, biological process and molecular function, respectively. In addition, the number of protein pairs with annotation overlap in two or three ontologies is relatively small (<112). Therefore, we may conclude that "significant associations" can be attributed to diverse functional relationships, such as forming protein complexes, binding to the same molecules, sharing similar functional activities, or simply participating in the same biological processes.

Appendix B: Analysis of GO-term predictions

By manual inspection, we found that the predicted GO annotations in the “biological process” generally had more favorable literature support than others in the “cellular component” and “biological function” (see S2 Table). More specifically, we found it difficult to accurately predict the GO terms that specify the identity of an interacting partner or annotations to well-defined small protein complexes. This was surprising: although the significant protein pairs have the most annotation overlap rate in the “cellular component” of protein complexes, predictions based on the significant pairs did not perform best when identifying complex members. This shows that, for GO, biological processes may be the best targets of our predictions. KEGG pathway predictions, similar to biological processes, should also give favorable error rates.

Appendix C: Analysis on the independence between the PPI dataset and the annotation datasets (GO and KEGG)

The KEGG pathways are based on more than 2000 publications, of which only 53 overlap the 21,372 publications of the human PPI network. These 53 publications are only associated with 273 PPIs (<1%) in the network. Thus, the KEGG pathway annotations are considered as a dataset independent of the human PPI dataset. For the GO annotations, however, ~19% of the supporting publications overlap the PPI literature, which are associated with over 20% of all the PPIs. Although the GO annotations are not necessarily derived from or highly correlated with the PPIs in the same publication, such a literature overlap rate at least suggests that the GO annotations are not as an independent tool as the KEGG annotations when being used to assess the functional associations in the human PPI network.
