# Supplementary material for: PAND: A Distribution to Identify Functional Linkage from Networks with Preferential Attachment Property
Source: PLoS One. 2015 Jul 9;10(7):e0127968. doi: 10.1371/journal.pone.0127968 (PMC4497646; doi:10.1371/journal.pone.0127968)
Supplement: S2 File — (PDF) [file pone.0127968.s012.pdf]

User manual for PANDA: Preferential Attachment based common Neighbor Distribution derived functional Associations

Hua Li and Pan Tong

2014-10-30

Contents

1 Introduction **1**

2 A Quick Example **1**

3 File Location and Session Info **4**

1 Introduction

This file gives a brief introduction on the functions used in the R package of PANDA (preferential-attachment based common neighbor distribution derived associations). PANDA is designed to perform the following tasks in protein-protein interaction (PPI) networks: (1) identify significantly functionally associated protein pairs, (2) predict GO terms and KEGG pathways for proteins, (3) make a cluster of proteins based on the significant protein pairs, (4) identify subclusters whose members are enriched in KEGG pathways. For other types of biological networks, (1) and (3) can still be performed. For more details on PANDA, please refer to "PAND: a distribution to identify functional linkage from networks with preferential attachment property", or consult Hua Li (kaixinsjtu@hotmail.com).

2 A Quick Example

The first step is to load the package from the library.

```
> library(PANDA)
```

Then we load the example data shipped with this package.

```
> data(dfPPI)
> data(GENE2GOtopLite)
```

```
> data(GENE2KEGG)
> data(KEGGID2NAME)
```

The “dfPPI” is a PPI network consisting of 2360 proteins and 5355 interactions that is used as an example to demonstrate the capability of PANDA. The “GENE2GOtopLite” and “GENE2KEGG” are examples of GO and KEGG annotations of proteins. “KEGGID2NAME” maps KEGG pathway ID to KEGG pathway names.

We first apply PAND to the PPI network to derive functional links between protein by using the function “SignificantPairs” (protein pairs will be ranked by p-values if ”pvalue=TRUE” is specified, otherwise by probabilities):

```
> OrderAll=SignificantPairs(PPIdb=dfPPI)
> head(OrderAll)
```

```
        Sym_A   Sym_B Probability CommonNeighbor
10234   CPNE1   CPNE4   -69.52455             18
8343  SMARCA4 SMARCA2   -54.93328             26
26211    LDB1    LDB2   -47.30440             16
1       SMAD2   SMAD3   -39.21016             20
4255     PER1    PER2   -39.00400             10
21882  JARID2    MTF2   -38.85149             10
```

Based on the p-values (or probabilities) of the significant protein pairs obtained above, we can perform agglomerative hierarchical clustering (using the unweighted group average) for proteins of all significant pairs.This function returns an object in the class “dendrogram”. If “Plot=TRUE” is specified, it will also plot the dendrogram.

```
> dendMap=ProteinCluster(Pfile=OrderAll, Plot=TRUE, TextScaler=50)
```

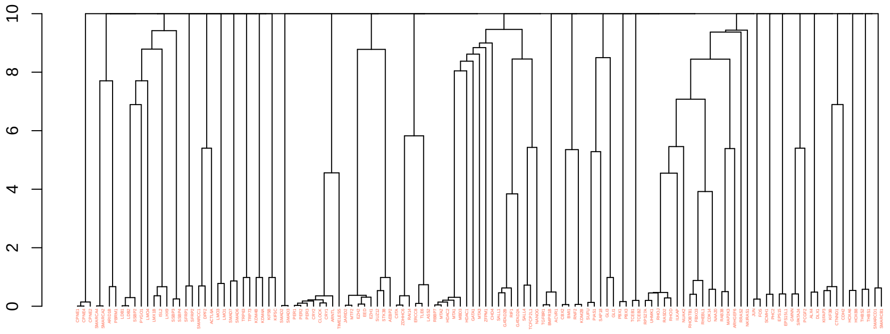

The significant protein pairs generated by the function “SignificantPairs” constituted a new network, with which we can make further functional predictions. We use the functions “GOpredict” and “KEGGpredict” to perform functional enrichment analysis (p-values were calculated with Fisher’s exact test) among a protein’s significant partners to predict GO terms and KEGG pathways for the protein:

```
> GP=GOpredict(Pfile=OrderAll, PPIdb=dfPPI, Gene2Annotation=GENE2GOtopLite, p_value=0.001)
> head(GP)
```

```
   Symbol       GOID
1  TGFBR1 GO:0045669
2    CBX2 GO:0071535
3    MTA1 GO:0016581
4    PBX1 GO:0007387
5   THBS2 GO:0048603
6 GATAD2A GO:0072092
                                           GOterm Ratio
1 positive regulation of osteoblast differentiation   2/2
2             RING-like zinc finger domain binding   2/2
3                                      NuRD complex   4/5
4            anterior compartment pattern formation   1/1
5                   fibroblast growth factor binding   1/1
6                             ureteric bud invasion   1/2
        Pvalue
1 5.496440e-05
2 3.592444e-07
3 2.764488e-09
4 4.237288e-04
5 4.237288e-04
6 8.474576e-04
```

```
> KP=KEGGpredict(Pfile=OrderAll, PPIdb=dfPPI, Gene2Annotation=GENE2KEGG,
    p_value=0.001, IDtoNAME=KEGGID2NAME)
> head(KP)
```

```
    Symbol   KEGGID                  PathName Ratio       Pvalue
1    SAP18 hsa05217      Basal cell carcinoma   2/2 5.334780e-04
2 TIMELESS hsa04710 Circadian rhythm - mammal   4/4 5.545925e-10
```

We use the following function to identify subclusters (from the cluster generated by “ProteinCluster”) whose members are significantly enriched in any KEGG pathway (if KGremove=TURE, “hsa05200” and “hsa01100” will be excluded from this analysis as they are too broad):

```
> SignificantSubcluster(Dendrogram=dendMap, Gene2Annotation=GENE2KEGG,
   PPIdb=dfPPI, KGremove=TRUE, SPoint=1, EPoint=9.7)
```

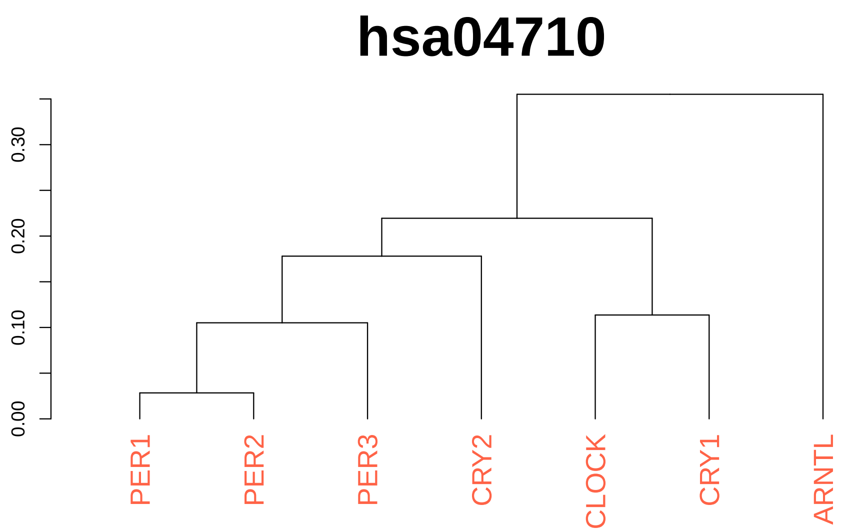

3 File Location and Session Info

```
> sessionInfo()
```

```
R version 3.1.2 (2014-10-31)
Platform: x86_64-apple-darwin13.4.0 (64-bit)

locale:
[1] C/en_US.UTF-8/en_US.UTF-8/C/en_US.UTF-8/en_US.UTF-8

attached base packages:
[1] parallel  stats     graphics  grDevices utils     datasets
[7] methods   base

other attached packages:
[1] PANDA_0.9.9          AnnotationDbi_1.26.1 GenomeInfoDb_1.0.2
[4] Biobase_2.24.0       BiocGenerics_0.10.0  RSQLite_1.0.0
[7] DBI_0.3.1

loaded via a namespace (and not attached):
[1] GO.db_2.14.0    IRanges_1.22.10 cluster_1.15.3  stats4_3.1.2
[5] tools_3.1.2
```
