# Supplementary material for: PAND: A Distribution to Identify Functional Linkage from Networks with Preferential Attachment Property
Source: PLoS One. 2015 Jul 9;10(7):e0127968. doi: 10.1371/journal.pone.0127968 (PMC4497646; doi:10.1371/journal.pone.0127968)
Supplement: S2 Table — For each protein, the ratio shows the number of significant partners (denominator) and the number of significant partners with the assigned GO/KEGG annotation (numerator). P-values were calculated by Fisher’s exact test based on the annotations of all significant partners for each protein. (DOCX) [file pone.0127968.s015.docx]

S2 Table. Predictions of GO and KEGG pathway annotations.

| **Protein** | **GO ID** | **GO term** | **p-value** | **Inspection Result** |
| --- | --- | --- | --- | --- |
| **MED10** | GO:0016592 | mediator complex | 2.54E-44 | supported |
| **MED19** | GO:0016592 | mediator complex | 1.42E-44 | supported |
| **MED9** | GO:0016592 | mediator complex | 7.78E-45 | supported |
| **MED28** | GO:0016592 | mediator complex | 7.78E-45 | supported |
| **JAK2** | GO:0005159 | insulin-like growth factor receptor binding | 2.28E-12 | likely |
| **SHC1** | GO:0005070 | SH3/SH2 adaptor activity | 2.53E-18 | supported |
| **STK24** | GO:0000159 | protein phosphatase type 2A complex | 1.70E-15 | unlikely |
| **IRS1** | GO:0043560 | insulin receptor substrate binding | 1.13E-13 | unlikely |
| **NCOA1** | GO:0016922 | ligand-dependent nuclear receptor binding | 4.63E-12 | supported |
| **CASP10** | GO:0008633 | activation of pro-apoptotic gene products | 1.44E-21 | supported |
| **MOBKL3** | GO:0000159 | protein phosphatase type 2A complex | 9.68E-16 | unlikely |
| **FAM40A** | GO:0000159 | protein phosphatase type 2A complex | 4.29E-13 | unlikely |
| **CTTNBP2** | GO:0000159 | protein phosphatase type 2A complex | 2.21E-15 | unlikely |
| **RBBP7** | GO:0016580 | Sin3 complex | 3.00E-13 | supported |
| **PLCG1** | GO:0005070 | SH3/SH2 adaptor activity | 1.23E-19 | likely |
| **PIK3R1** | GO:0005070 | SH3/SH2 adaptor activity | 2.61E-16 | supported |
| **IGF1R** | GO:0005159 | insulin-like growth factor receptor binding | 2.92E-11 | unlikely |
| **PDCD10** | GO:0000159 | protein phosphatase type 2A complex | 1.95E-13 | unlikely |
| **CTTNBP2NL** | GO:0000159 | protein phosphatase type 2A complex | 3.34E-13 | unlikely |
| **SMAD3** | GO:0070411 | I-SMAD binding | 5.84E-12 | unlikely |
| **JAK3** | GO:0005159 | insulin-like growth factor receptor binding | 1.72E-11 | unlikely |
| **MSN** | GO:0008633 | activation of pro-apoptotic gene products | 6.36E-17 | likely |
| **TAF1** | GO:0033276 | transcription factor TFTC complex | 4.64E-21 | unlikely |
| **SYK** | GO:0005070 | SH3/SH2 adaptor activity | 3.62E-12 | supported |
| **CCT8** | GO:0000159 | protein phosphatase type 2A complex | 1.47E-13 | unlikely |
| **STK25** | GO:0000159 | protein phosphatase type 2A complex | 7.89E-14 | unlikely |
| **TAF11** | GO:0033276 | transcription factor TFTC complex | 4.64E-21 | unlikely |
| **MKRN3** | GO:0051865 | protein autoubiquitination | 2.83E-11 | likely |
| **SIKE1** | GO:0000159 | protein phosphatase type 2A complex | 3.50E-11 | unlikely |
| **RP6-213H19.1** | GO:0000159 | protein phosphatase type 2A complex | 3.95E-14 | unlikely |
| **TCP1** | GO:0000159 | protein phosphatase type 2A complex | 7.17E-16 | unlikely |
| **POLR2D** | GO:0016592 | mediator complex | 7.86E-34 | unlikely |
| **SLMAP** | GO:0000159 | protein phosphatase type 2A complex | 2.00E-11 | unlikely |
| **DTX3L** | GO:0051865 | protein autoubiquitination | 1.98E-11 | likely |
| **SNRPD1** | GO:0005683 | U7 snRNP | 4.63E-11 | unlikely |
| **UBOX5** | GO:0051865 | protein autoubiquitination | 1.05E-11 | likely |
| **RNF114** | GO:0051865 | protein autoubiquitination | 1.36E-11 | likely |
| **TAF8** | GO:0033276 | transcription factor TFTC complex | 3.58E-22 | unlikely |
| **DDX20** | GO:0005683 | U7 snRNP | 7.96E-14 | unlikely |
| **TRIM2** | GO:0051865 | protein autoubiquitination | 1.75E-11 | unlikely |
| **RBP1** | GO:0016580 | Sin3 complex | 3.85E-14 | supported |
| **DTX3** | GO:0051865 | protein autoubiquitination | 1.55E-11 | supported |
| **FAM40B** | GO:0000159 | protein phosphatase type 2A complex | 1.08E-13 | unlikely |
| **MID1** | GO:0051865 | protein autoubiquitination | 1.36E-11 | likely |
| **RARA** | GO:0010887 | negative regulation of cholesterol storage | 5.05E-11 | Supported |
| **COL4A1** | GO:0005587 | collagen type IV | 4.87E-17 | supported |
| **MED7** | GO:0016592 | mediator complex | 2.61E-47 | supported |
| **SAP30** | GO:0016580 | Sin3 complex | 3.85E-14 | supported |
| **TNFRSF1A** | GO:0008633 | activation of pro-apoptotic gene products | 3.13E-19 | supported |
| **UBE2N** | GO:0070936 | protein K48-linked ubiquitination | 1.21E-14 | unlikely |
| **SNRPD2** | GO:0005683 | U7 snRNP | 7.96E-14 | unlikely |
| **MED25** | GO:0016592 | mediator complex | 1.11E-47 | supported |
| **RPAP2** | GO:0005665 | DNA-directed RNA polymerase II, core complex | 4.63E-11 | supported |
| **TAF13** | GO:0033276 | transcription factor TFTC complex | 3.58E-22 | unlikely |
| **MED23** | GO:0016592 | mediator complex | 1.11E-47 | supported |
| **VAV1** | GO:0005070 | SH3/SH2 adaptor activity | 1.04E-16 | supported |
| **MBD3L1** | GO:0016581 | NuRD complex | 1.43E-15 | supported |
| **LCP2** | GO:0005070 | SH3/SH2 adaptor activity | 1.56E-14 | supported |
| **RNF125** | GO:0051865 | protein autoubiquitination | 1.75E-11 | supported |
| **TBP** | GO:0033276 | transcription factor TFTC complex | 9.06E-20 | unlikely |
| **SOCS1** | GO:0005158 | insulin receptor binding | 1.80E-11 | unlikely |
| **TRIM39** | GO:0051865 | protein autoubiquitination | 3.83E-12 | unlikely |
| **SIN3A** | GO:0016581 | NuRD complex | 2.26E-14 | supported |
| **RNF167** | GO:0051865 | protein autoubiquitination | 1.36E-11 | supported |
| **UBE2W** | GO:0070936 | protein K48-linked ubiquitination | 6.78E-22 | unlikely |
| **BRMS1** | GO:0016580 | Sin3 complex | 1.63E-11 | supported |
| **CBL** | GO:0005070 | SH3/SH2 adaptor activity | 4.28E-20 | supported |
| **APC2** | GO:0005680 | anaphase-promoting complex | 5.43E-25 | supported |
| **EFTUD2** | GO:0005682 | U5 snRNP | 9.89E-11 | supported |
| **RASA1** | GO:0042169 | SH2 domain binding | 1.39E-12 | likely |
| **POLR2F** | GO:0016592 | mediator complex | 7.66E-42 | unlikely |
| **PPP4C** | GO:0000159 | protein phosphatase type 2A complex | 8.01E-13 | unlikely |
| **BIRC8** | GO:0051865 | protein autoubiquitination | 9.15E-12 | unlikely |
| **ARHGDIA** | GO:0008633 | activation of pro-apoptotic gene products | 6.36E-17 | likely |
| **PSMD4** | GO:0070628 | proteasome binding | 8.17E-12 | unlikely |
| **USP22** | GO:0030914 | STAGA complex | 1.44E-14 | supported |
| **MBD2** | GO:0016581 | NuRD complex | 2.35E-17 | supported |
| **CDC20** | GO:0005680 | anaphase-promoting complex | 5.51E-28 | supported |
| **POLR2K** | GO:0005665 | DNA-directed RNA polymerase II, core complex | 1.06E-27 | supported |
| **VAMP2** | GO:0042581 | specific granule | 9.25E-11 | unlikely |
| **GEMIN6** | GO:0005683 | U7 snRNP | 8.84E-11 | unlikely |
| **RNF185** | GO:0051865 | protein autoubiquitination | 2.24E-11 | likely |
| **TRAF3IP3** | GO:0000159 | protein phosphatase type 2A complex | 1.47E-13 | unlikely |
| **INHBB** | GO:0032927 | positive regulation of activin receptor signaling pathway | 9.89E-11 | supported |
| **POLR3C** | GO:0003709 | RNA polymerase III transcription factor activity | 2.19E-16 | unlikely |
| **MED15** | GO:0016592 | mediator complex | 1.74E-48 | supported |
| **MAP2K7** | GO:0005078 | MAP-kinase scaffold activity | 1.98E-11 | likely |
| **IRS2** | GO:0005159 | insulin-like growth factor receptor binding | 8.91E-11 | likely |
| **MED13L** | GO:0016592 | mediator complex | 2.74E-46 | supported |
| **EZR** | GO:0008633 | activation of pro-apoptotic gene products | 2.22E-12 | supported |
| **UBE2U** | GO:0070936 | protein K48-linked ubiquitination | 5.23E-23 | likely |
| **CDC2L6** | GO:0016592 | mediator complex | 1.40E-40 | supported |
| **CHTF18** | GO:0006297 | nucleotide-excision repair, DNA gap filling | 1.69E-16 | supported |
| **KRT1** | GO:0001533 | cornified envelope | 3.59E-11 | likely |
| **RFWD2** | GO:0000159 | protein phosphatase type 2A complex | 6.48E-11 | unlikely |
| **MEN1** | GO:0042800 | histone methyltransferase activity (H3-K4 specific) | 1.02E-15 | Supported |
| **POP4** | GO:0005655 | nucleolar ribonuclease P complex | 3.24E-13 | supported |
| **EIF1B** | GO:0003743 | translation initiation factor activity | 5.22E-18 | supported |
| **MED31** | GO:0016592 | mediator complex | 2.18E-25 | supported |
| **HCFC2** | GO:0042800 | histone methyltransferase activity (H3-K4 specific) | 2.72E-15 | Supported |
| **RPP25** | GO:0005655 | nucleolar ribonuclease P complex | 3.24E-13 | supported |
| **GDF5** | GO:0060395 | SMAD protein signal transduction | 3.89E-13 | supported |
| **CCNC** | GO:0016592 | mediator complex | 2.64E-33 | supported |
| **GTF2F2** | GO:0005665 | DNA-directed RNA polymerase II, core complex | 8.82E-13 | unlikely |
| **DSC1** | GO:0030057 | desmosome | 4.54E-12 | supported |
| **PTK2B** | GO:0042169 | SH2 domain binding | 6.97E-12 | supported |
| **TAF15** | GO:0033276 | transcription factor TFTC complex | 3.96E-20 | unlikely |
| **TGFBR2** | GO:0005114 | type II transforming growth factor beta receptor binding | 1.10E-16 | unlikely |
| **SETD1A** | GO:0042800 | histone methyltransferase activity (H3-K4 specific) | 1.02E-15 | supported |
| **CTNND1** | GO:0071681 | cellular response to indole-3-methanol | 1.39E-13 | Supported |
| **ATXN7L3** | GO:0030914 | STAGA complex | 3.85E-14 | supported |
| **PLCG2** | GO:0005070 | SH3/SH2 adaptor activity | 4.78E-12 | supported |
| **PTPN1** | GO:0005158 | insulin receptor binding | 6.41E-11 | likely |
| **PPP2R1B** | GO:0000159 | protein phosphatase type 2A complex | 2.71E-14 | supported |
| **PI3** | GO:0001533 | cornified envelope | 3.59E-11 | likely |
| **FAM175A** | GO:0070552 | BRISC complex | 7.91E-11 | unlikely |
| **KLF1** | GO:0071564 | npBAF complex | 2.14E-11 | unlikely |
| **DCAF11** | GO:0031464 | Cul4A-RING ubiquitin ligase complex | 2.14E-11 | supported |
| **RPP21** | GO:0005655 | nucleolar ribonuclease P complex | 4.94E-11 | supported |
| **IGBP1** | GO:0000159 | protein phosphatase type 2A complex | 2.23E-12 | unlikely |
| **TNFRSF10A** | GO:0008633 | activation of pro-apoptotic gene products | 1.06E-13 | supported |
| **TGFB2** | GO:0050431 | transforming growth factor beta binding | 1.31E-15 | supported |
| **MTA1** | GO:0016581 | NuRD complex | 1.99E-11 | supported |
| **CCT6A** | GO:0000159 | protein phosphatase type 2A complex | 3.50E-11 | unlikely |
| **FGFR1OP2** | GO:0000159 | protein phosphatase type 2A complex | 1.48E-11 | unlikely |
| **BRMS1L** | GO:0016580 | Sin3 complex | 9.73E-14 | supported |
| **UBE2V1** | GO:0070936 | protein K48-linked ubiquitination | 2.09E-22 | unlikely |
| **MED27** | GO:0016592 | mediator complex | 1.12E-45 | supported |
| **USP3** | GO:0003743 | translation initiation factor activity | 1.41E-21 | unlikely |
| **GATAD2B** | GO:0016581 | NuRD complex | 4.63E-11 | likely |
| **TADA1L** | GO:0030914 | STAGA complex | 9.18E-13 | supported |
| **TRPC7** | GO:0015279 | store-operated calcium channel activity | 7.91E-11 | supported |
| **Protein** | **KEGG ID** | **KEGG pathway name** | **p-value** |  |
| **MED29** | hsa03020 | RNA polymerase | 1.22E-22 | unlikely |
| **MED10** | hsa03020 | RNA polymerase | 1.69E-22 | unlikely |
| **MED19** | hsa03020 | RNA polymerase | 1.22E-22 | unlikely |
| **MED9** | hsa03020 | RNA polymerase | 2.06E-20 | unlikely |
| **MED28** | hsa03020 | RNA polymerase | 2.06E-20 | unlikely |
| **PTPN6** | hsa04664 | Fc epsilon RI signaling pathway | 1.13E-15 | likely |
| **LYN** | hsa04650 | Natural killer cell mediated cytotoxicity | 4.71E-15 | likely |
| **MED26** | hsa03020 | RNA polymerase | 1.22E-22 | unlikely |
| **UCHL5** | hsa03050 | Proteasome | 8.12E-36 | supported |
| **IGF1R** | hsa04012 | ErbB signaling pathway | 4.56E-16 | supported |
| **PAAF1** | hsa03050 | Proteasome | 1.16E-31 | supported |
| **PSMD10** | hsa03050 | Proteasome | 2.10E-33 | supported |
| **SRC** | hsa04062 | Chemokine signaling pathway | 2.86E-14 | unlikely |
| **MSN** | hsa04210 | Apoptosis | 2.12E-14 | likely |
| **INSR** | hsa04630 | Jak-STAT signaling pathway | 1.12E-17 | unlikely |
| **SUPT3H** | hsa03022 | Basal transcription factors | 2.56E-14 | unlikely |
| **BCAR1** | hsa04012 | ErbB signaling pathway | 5.05E-11 | supported |
| **TAF8** | hsa03022 | Basal transcription factors | 6.01E-31 | supported |
| **GAB2** | hsa04650 | Natural killer cell mediated cytotoxicity | 5.82E-16 | supported |
| **RPAP2** | hsa03020 | RNA polymerase | 2.16E-12 | supported |
| **LCP2** | hsa04662 | B cell receptor signaling pathway | 6.36E-18 | likely |
| **KIT** | hsa05220 | Chronic myeloid leukemia | 1.90E-15 | likely |
| **CBL** | hsa04650 | Natural killer cell mediated cytotoxicity | 2.40E-22 | supported |
| **APC2** | hsa04110 | Cell cycle | 2.03E-19 | supported |
| **RASA1** | hsa04650 | Natural killer cell mediated cytotoxicity | 4.09E-12 | likely |
| **ARHGDIA** | hsa04210 | Apoptosis | 2.12E-14 | Likely |
| **ENG** | hsa04350 | TGF-beta signaling pathway | 6.83E-12 | supported |
| **GIYD2** | hsa03050 | Proteasome | 1.89E-16 | Likely |
| **INPP5D** | hsa05220 | Chronic myeloid leukemia | 1.17E-11 | Likely |
| **GAB1** | hsa05220 | Chronic myeloid leukemia | 5.25E-17 | Likely |
| **BLNK** | hsa04664 | Fc epsilon RI signaling pathway | 1.41E-12 | likely |
| **IRS2** | hsa04630 | Jak-STAT signaling pathway | 1.76E-19 | supported |
| **PAG1** | hsa04650 | Natural killer cell mediated cytotoxicity | 4.18E-17 | likely |
| **MED13L** | hsa03020 | RNA polymerase | 3.97E-12 | unlikely |
| **EZR** | hsa04210 | Apoptosis | 2.34E-11 | supported |
| **NAPA** | hsa04130 | SNARE interactions in vesicular transport | 2.61E-12 | supported |
| **CHTF18** | hsa03430 | Mismatch repair | 1.38E-15 | likely |
| **SH3BP2** | hsa04660 | T cell receptor signaling pathway | 3.79E-15 | supported |
| **USP14** | hsa03050 | Proteasome | 8.12E-36 | supported |
| **PTK2B** | hsa04012 | ErbB signaling pathway | 6.36E-13 | likely |
| **TAF15** | hsa03022 | Basal transcription factors | 3.32E-23 | supported |
| **ADRM1** | hsa03050 | Proteasome | 4.99E-13 | supported |
| **SHB** | hsa04660 | T cell receptor signaling pathway | 3.17E-13 | supported |
| **SH2B2** | hsa04630 | Jak-STAT signaling pathway | 5.03E-13 | supported |
| **PTPN1** | hsa04722 | Neurotrophin signaling pathway | 6.94E-11 | likely |
| **NFKB2** | hsa04660 | T cell receptor signaling pathway | 2.86E-12 | supported |
| **ITK** | hsa04650 | Natural killer cell mediated cytotoxicity | 2.02E-15 | supported |
| **MCM10** | hsa04110 | Cell cycle | 1.89E-24 | supported |
| **CDT1** | hsa04110 | Cell cycle | 1.12E-11 | supported |
| **UBE2V1** | hsa04120 | Ubiquitin mediated proteolysis | 1.24E-20 | unlikely |
| **MED27** | hsa03020 | RNA polymerase | 5.98E-12 | unlikely |
| **MAP4K1** | hsa04664 | Fc epsilon RI signaling pathway | 2.58E-11 | likely |

For each protein, the ratio shows the number of significant partners (denominator) and the number of significant partners with the assigned GO/KEGG annotation (numerator). P-values were calculated by Fisher’s exact test based on the annotations of all significant partners for each protein.
