# Supplementary material for: PAND: A Distribution to Identify Functional Linkage from Networks with Preferential Attachment Property
Source: PLoS One. 2015 Jul 9;10(7):e0127968. doi: 10.1371/journal.pone.0127968 (PMC4497646; doi:10.1371/journal.pone.0127968)
Supplement: S3 Table — Each row corresponds to a subcluster in Fig 3 with the same KEGG ID. The 1st column (Protein) lists the proteins without the KEGG annotation in the 2nd column. Ratio equals the percentage of proteins with the same KEGG annotation within the subcluster; height equals the level at which the subcluster was obtained. P-values were calculated with Fisher’s exact test for each subcluster. (DOCX) [file pone.0127968.s016.docx]

S3 Table. Functional inferences based on our clustering scheme.

| **Protein** | **KEGG ID** | **KEGG pathway name** | **Ratio** | **P-value** | **Height** |
| --- | --- | --- | --- | --- | --- |
| UBE2V1, UBE2V2 | hsa04120 | Ubiquitin mediated proteolysis | 0.882 | 2.59E-27 | 7.9 |
| TAF8, TAF15, SUPT3H, USP22, KAT2A, TRRAP, ATXN7, ATXN7L3, ENY2, SUPT7L, TADA1L | hsa03022 | Basal transcription factors | 0.607 | 7.27E-37 | 8.8 |
| None | hsa03020 | RNA polymerase | 1 | 1.15E-32 | 2.3 |
| MSN, EZR, ARHGDIA | hsa04210 | Apoptosis | 0.8 | 2.35E-23 | 6.1 |
| CXCR4, PTPN2, PDGFRB, PDGFRA, KDR, FLT1, IRS1, IRS2, IGF1R, INSR, BCR, ABL1, MST1R, IRS4, INPP5D, KIT, SHC1, PLCG1, CRK, CRKL, GAB2, GAB1, SYK, ZAP70, VAV1, LCP2, LAT, SH3BP2, CD19, SHB, FYN, LCK, LYN, HCK, SRC, YES1, BLNK, MAP4K1, BTK, PLCG2, TEC, GRAP, PAG1, SIT1, LAX1, CD22, KHDRBS1, ITK, PECAM1, DAPP1, PTK2, PTK2B, PXN, BCAR1, NEDD9, CSK, PTPN12, RASA1, DOK1, DOK2, DOK3, SH2B2, SH2B1 | hsa04630 | Jak-STAT signaling pathway | 0.284 | 4.33E-26 | 9.2 |
| CHTF18, CHTF8, DSCC1, BRD4, CDKN1A, RAD17 | hsa03030 | DNA replication | 0.625 | 3.83E-22 | 9.7 |
| None | hsa04110 | Cell cycle | 1 | 1.75E-32 | 1 |
| UCHL5, USP14, PSMD10, PAAF1, ADRM1 | hsa03050 | Proteasome | 0.75 | 8.36E-33 | 5.9 |
| APC2 | hsa04110 | Cell cycle | 0.929 | 2.61E-25 | 8 |
| None | hsa05218 | Melanoma | 1 | 5.04E-25 | 6 |
| GIYD2, PLK1 | hsa03050 | Proteasome | 0.857 | 1.40E-27 | 9.6 |

Each row corresponds to a subcluster in Fig. 4 with the same KEGG ID. The 1^st^ column (Protein) lists the proteins without the KEGG annotation in the 2^nd^ column. Ratio= the percentage of proteins with the same KEGG annotation within the subcluster; height = level at which the subcluster was obtained. P-values were calculated with Fisher’s exact test for each subcluster.
