# Supplementary material for: PAND: A Distribution to Identify Functional Linkage from Networks with Preferential Attachment Property
Source: PLoS One. 2015 Jul 9;10(7):e0127968. doi: 10.1371/journal.pone.0127968 (PMC4497646; doi:10.1371/journal.pone.0127968)

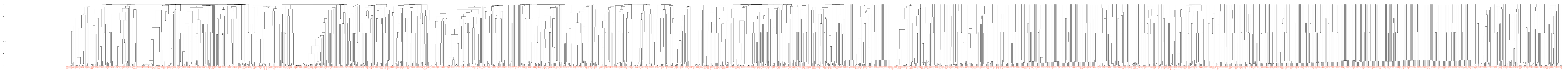

Supplement: S6 Fig — The bar on the left side indicates the height in the cluster. (PDF) [file pone.0127968.s006.pdf]
